# Building an emission library of donor–acceptor–donor type linker-based luminescent metal–organic frameworks†

**DOI:** 10.1039/d2sc02267b

**Published:** 2022-06-17

**Authors:** Hai-Lun Xia, Kang Zhou, Shenjie Wu, Daming Ren, Kai Xing, Jiandong Guo, Xiaotai Wang, Xiao-Yuan Liu, Jing Li

**Affiliations:** Hoffmann Institute of Advanced Materials, Shenzhen Polytechnic 7098 Liuxian Blvd, Nanshan District Shenzhen 518055 People's Republic of China liuxiaoyuan1989@szpt.edu.cn; Department of Chemistry, College of Basic Medicine, Third Military Medical University (Army Medical University) Chongqing 400038 People's Republic of China; Department of Chemistry, University of Colorado Denver Campus Box 194, P. O. Box 173364 Denver Colorado 80217-3364 USA; Department of Chemistry and Chemical Biology, Rutgers University 123 Bevier Road, Piscataway New Jersey 08854 USA jingli@rutgers.edu

## Abstract

Luminescent metal–organic frameworks (LMOFs) have been extensively studied for their potential applications in lighting, sensing and biomedicine-related areas due to their high porosity, unlimited structure and composition tunability. However, methodical development in systematically tuning the emission properties of fluorescent organic linker-based LMOFs to facilitate the rational design and synthesis of target-specific materials has remained challenging. Herein we attempt to build an emission library by customized synthesis of LMOFs with targeted absorption and emission properties using donor–acceptor–donor type organic linkers. By tuning the acceptor groups (*i.e.* 2,1,3-benzothiadiazole and its derivatives), donor groups (including modification of original donors and use of donors with different metal–linker connections) and bridging units between acceptor and donor groups, an emission library is developed for LMOFs with their emissions covering the entire visible light range as well as the near-infrared region. This work may offer insight into well controlled design of organic linkers for the synthesis of LMOFs with specified functionality.

## Abstract 2

An emission library was built for donor–acceptor–donor type linker-based LMOFs, which can be used to rationally design organic linkers to prepare LMOFs with emission from deep blue to near-infrared.

## Graphical abstract

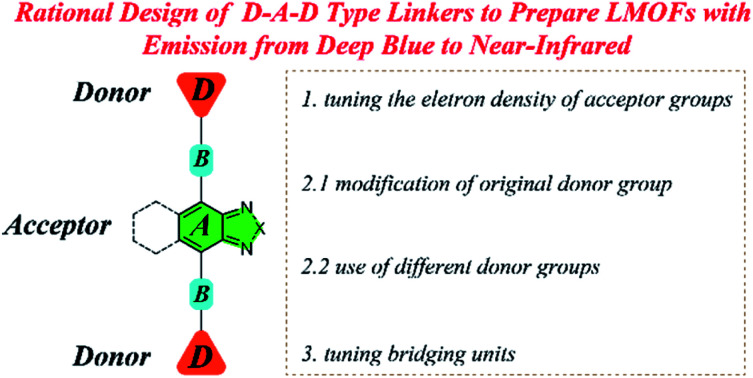

## Introduction

Organic linker-based luminescent metal–organic frameworks (LMOFs)^1,2^ have shown great potential for applications in various areas, such as solid-state-lighting,^3–7^ sensing,^8–12^ and bioimaging^13,14^ due to their highly tunable structures and compositions by varying organic linkers and metal nodes and *via* crystal engineering under various conditions. To tune the emission properties of LMOFs in a broad energy region (including the visible light and near-infrared range) and to study the structural effect on their emission behaviors, it is essential to build design principles based on the structure–property relationship, which will facilitate the customized synthesis and target-specific applications of LMOFs. However, tuning the emission behaviors of organic linker based LMOFs in a fully controllable manner remains a challenging task.

In our previous work, we achieved full-color emissive LMOFs using 2,1,3-benzothiadiazole and its derivative-based dicarboxylic acids and tetratopic carboxylic acids as organic linkers.^15,16^ In these donor–acceptor–donor (D–A–D) based linkers, the emission tunability relies on the changeable electron-withdrawing capacity of acceptor groups. While these acceptors have been used in different D–A–D linkers to prepare LMOFs for various applications,^10,17–23^ a systematic study to tune the emission properties of D–A–D linker based LMOFs and to develop a related design principle is still lacking.

**Scheme 1:**
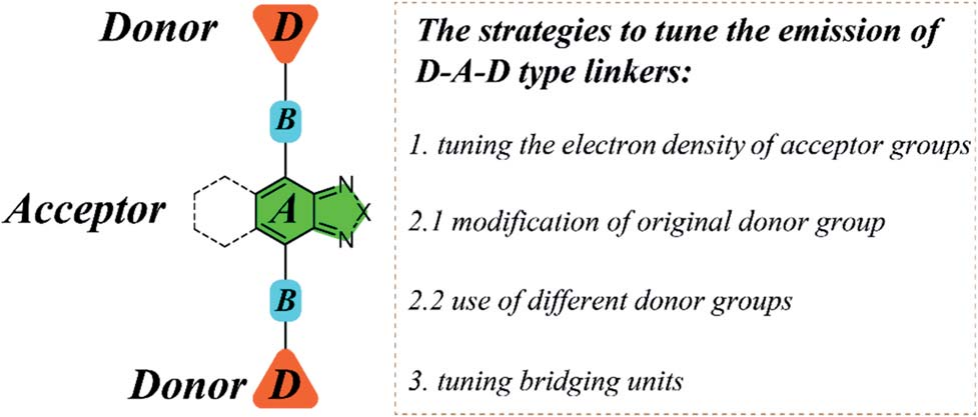
The schematic diagram illustrating the structure and multiple strategies to tune the emission properties of D–A–D type organic linkers using 2,1,3-benzothiadiazole and its derivative as acceptors.

For D–A–D type molecular linkers, three common strategies can be utilized to tune the emission behavior of LMOFs: (1) tuning the electron density of the acceptor groups; (2) tuning the electron density of the donor groups^15,16^ and (3) tuning the bridging units between donor and acceptor groups^24,25^ (Scheme 1). There are usually two approaches to changing the electron density of the donor groups: (2.1) modification of the original donor, such as addition of functional groups (*i.e.* NH_2_, OH, and CF_3_) with strong electron donating or electron withdrawing capacity; (2.2) use of totally different donor groups, for which metals may link to different binding sites, *e.g.* to oxygen when using carboxylic acid-based donor groups or nitrogen when using pyridine or azole-based donor groups (Scheme 1). Note that changing the electron density of acceptor groups usually has little or no effect on the topology of the resultant MOFs.^15,16^ However, changing the electron density of donor groups, which coordinate to the metal-containing clusters or ions to eventually form MOFs, often leads to structure variations.

Based on these strategies, we carry out a systematic study in the present work to tune the emission properties of D–A–D type linker based LMOFs by tailoring the acceptor groups, donor groups and the bridging units, where 2,1,3-benzothiadiazole and its derivative are utilized as the acceptor groups. Among the 23 organic linkers used in this study, 13 are newly synthesized or used to prepare MOFs for the first time, and 8 new MOF structures are obtained. For MOFs with the same structure as UiO-68, we name them UiO-68-L, in which L is the abbreviation of the organic linker; and for MOFs with new structures, we name them HIAM-*N* (HIAM = Hoffmann Institute of Advanced Materials), where *N* is the number for each new MOF. This relatively large database allows us to build an emission library with a broad range of emission energies, including both visible and near infrared (NIR) regions.

## Results and discussion

### Tuning acceptor groups

**Fig. 1:**
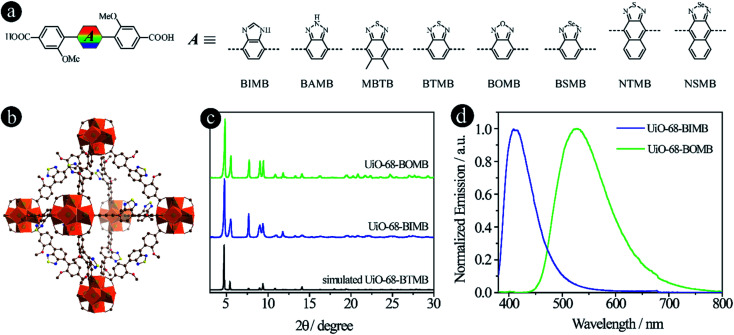
D–A–D type organic linkers with tunable acceptor groups. (a) The molecular structures of linkers with different acceptor groups. Among them, BAMB, MBTB, BTMB, BSMB, NTMB and NSMB were previously reported^15,21^ and BIMB and BOMB are newly synthesized; (b) the crystal structure of UiO-68-BTMB; (c) PXRD patterns of simulated UiO-68-BTMB and the as-synthesized UiO-68-BIMB and UiO-68-BOMB; (d) normalized solid-state emission spectra of UiO-68-BIMB and UiO-68-BOMB.

As reported in our previous work,^15^ the emission colors of LMOFs can be preciously controlled from blue to red by changing 2,1,3-benzothiadiazole and its derivative based acceptor groups from 2*H*-benzo[*d*][1,2,3]triazole, 5,6-dimethylbenzo[*c*][1,2,5]thiadiazole, benzo[*c*][1,2,5]thiadiazole, benzo[*c*][1,2,5]selenadiazole, naphtho[2,3-*c*][1,2,5]thiadiazole and naphtho[2,3-*c*][1,2,5]selenadiazole to form six dicarboxylic acids based linkers of 4,4′-(2*H*-benzo[*d*][1,2,3]triazole-4,7-diyl)bis(3-methoxybenzoic acid) (BAMB), 4,4′-(5,6-dimethylbenzo[*c*][1,2,5]thiadiazole-4,7-diyl)dibenzoic acid (MBTB), 4,4′-(benzo[*c*][1,2,5]thiadiazole-4,7-diyl)bis(3-methoxybenzoic acid) (BTMB), 4,4′-(benzo[*c*][1,2,5]selenadiazole-4,7-diyl)bis(3-methoxybenzoic acid) (BSMB), 4,4′-(naphtho[2,3-*c*][1,2,5]thiadiazole-4,9-diyl)bis(3-methoxybenzoic acid) (NTMB) and 4,4′-(naphtho[2,3-*c*][1,2,5]selenadiazole-4,9-diyl)bis(3-methoxybenzoic acid) (NSMB) (Fig. 1a). This strategy can be extended to other linker systems, such as tetratopic carboxylic acids, not only to realize tunable emission from blue to red, but also to increase the structural diversity of the resultant LMOFs with target properties.^16^

In an attempt to expand the emission library, two more acceptor groups of 1*H*-benzo[*d*]imidazole and benzo[*c*][1,2,5]oxadiazole were used to form 4,4′-(1*H*-benzo[*d*]imidazole-4,7-diyl)bis(3-methoxybenzoic acid) (BIMB) and 4,4′-(benzo[*c*][1,2,5]oxadiazole-4,7-diyl)bis(3-methoxybenzoic acid) (BOMB). These two linkers show deep blue and green emissions in *N*,*N*-dimethylformamide (DMF) solution (Fig. S1†), indicating that the electron density of benzo[*c*][1,2,5]oxadiazole is much lower than that of 1*H*-benzo[*d*]imidazole. Then two LMOFs, UiO-68-BIMB and UiO-68-BOMB, with the same topology as UiO-68-BTMB (Fig. 1b), were prepared according to the reported method.^15^ The PXRD patterns of these two LMOFs are almost identical to those of the simulated UiO-68-BTMB (Fig. 1c), confirming their isoreticular nature and high purity. UiO-68-BIMB and UiO-68-BOMB exhibit bright deep blue and green emission with the peak maximum at 417 nm and 530 nm, respectively. The corresponding photoluminescence quantum yield (PLQY) values are 69.5% and 5.9% under 365 nm excitation.

By tuning the acceptor groups, the emission energies of the resultant UiO-68-type LMOFs cover the entire visible light range (417, 445, 470, 520, 530, 545, 585 to 637 nm). These results further demonstrate that highly tunable emissions are achievable for D–A–D type organic linker based LMOFs by simply changing 2,1,3-benzothiadiazole and its derivative based acceptor groups.

### Modifying original donor groups

As mentioned earlier, two strategies can be used to tune the donor groups: (2.1) Modifying original donor groups, where the linker–metal bond model remains intact; (2.2) Employing different types of donor groups, in which new linker–metal bonds may form, leading to different structures.

**Fig. 2:**
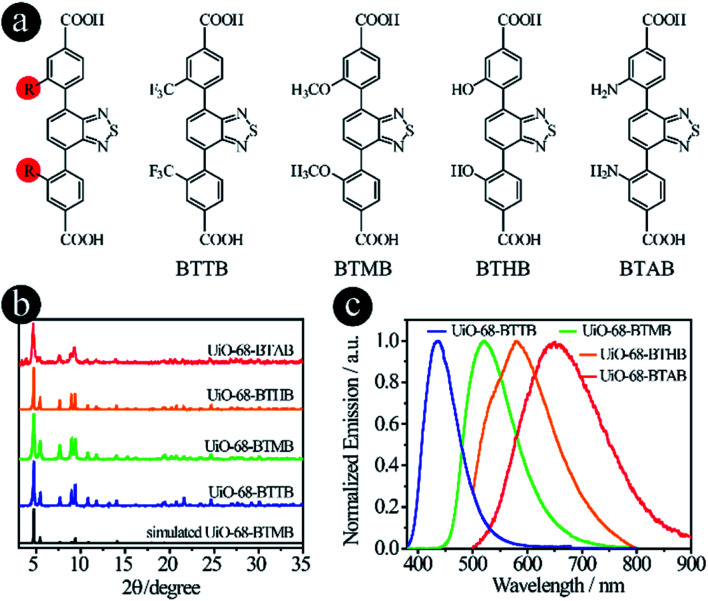
D–A–D type organic linkers with modified original donor groups. (a) The molecular structures of BTTB, BTMB, BTHB and BTAB; (b) the PXRD patterns and (c) the solid-state emission spectra of UiO-68-BTTB, UiO-68-BTMB, UiO-68-BTHB and UiO-68-BTAB.

To confirm that the modification of original donor groups indeed has the capacity to tune the emission of LMOFs, four linkers, 4,4′-(benzo[*c*][1,2,5]thiadiazole-4,7-diyl)bis(3-(trifluoromethyl)benzoic acid) (BTTB), 4,4′-(benzo[*c*]-[1,2,5]thiadiazole-4,7-diyl)bis(3-methoxybenzoic acid) (BTMB), 4,4′-(benzo[*c*][1,2,5]thiadiazole-4,7-diyl)bis(3-hydroxybenzoic acid) (BTHB) and 4,4′-(benzo[*c*][1,2,5]thiadiazole-4,7-diyl)bis(3-aminobenzoic acid) (BTAB), were designed and synthesized with single-site modification on the donor groups (Fig. 2a), where the order of the electron donating capacity is NH_2_ > OH > OCH_3_ > CF_3_. As expected, four UiO-68 type MOFs, UiO-68-BTTB, UiO-68-BTMB, UiO-68-BTHB and UiO-68-BTAB, were obtained, confirmed by the PXRD analysis (Fig. 2b). The solid-state emission of these LMOFs also covers the whole visible light spectrum from blue to red with the peak maximum at 438 nm, 520 nm, 575 nm and 650 nm for UiO-68-BTTB, UiO-68-BTMB, UiO-68-BTHB and UiO-68-BTAB, respectively (Fig. 2c). The corresponding PLQYs are 40.8%, 30.6%, 1.0% and 0.1%. It should be noted that a remarkable blue-shift, compared with UiO-68-BTMB, was observed when adding a group (such as CF_3_) with strong electron withdrawing capacity and a significant bathochromic shift was realized when using strong electron donating groups (*i.e.* OH and NH_2_).^26^ These results indicate that single-site modification of original donor groups is indeed a useful strategy to tune the emission properties of the LMOFs without changing the crystal structure.

### Tuning donor groups with different types of M–L bonds

Compared with carboxylic acid-based LMOFs, the utilization of donor groups with various metal–linker bonds will not only increase the structural diversity, but also introduce new or unprecedented properties to the resultant LMOFs. Herein, we prepared two series of organic linkers using pyridine and pyrazolate as the donor groups and investigated their effects on the emission behavior of the resultant LMOFs.

**Fig. 3:**
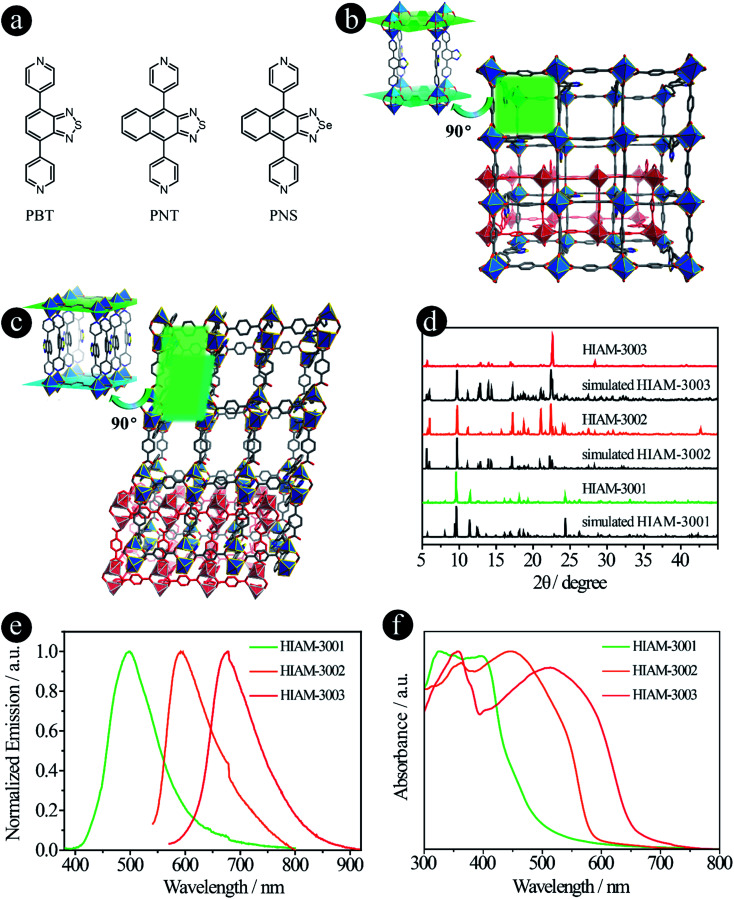
D–A–D type organic linkers with pyridine as the donor group. (a) The molecular structures of PBT, PNT and PNS; (b) the crystal structure of HIAM-3001; (c) the crystal structure of HIAM-3002; (d) the PXRD patterns of the simulated and as-synthesized HIAM-3001, HIAM-3002 and HIAM-3003; (e) the solid-state emission spectra and (f) the UV-vis absorption spectra of HIAM-3001, HIAM-3002 and HIAM-3003.

When pyridine was chosen as the donor group, three linkers, 4,7-di(pyridine-4-yl)benzo[*c*][1,2,5]thiadiazole (PBT), 4,9-di(pyridine-4-yl)naphtho[2,3-*c*][1,2,5]thiadiazole (PNT) and 4,9-di(pyridine-4-yl)naphtho[2,3-*c*][1,2,5]selenadiazole (PNS), were designed and synthesized (Fig. 3a). The emission wavelengths of PBT, PNT and PNS are 452 nm, 567 nm and 611 nm, respectively. Compared to their carboxylate counter parts BTMB (500 nm), NTMB (566 nm) and NSMB (610 nm) with the same acceptor groups, a significant blue-shift was observed for PBT (Fig. S2†), for which the acceptor group has a relative weak electron withdrawing capacity, like benzo[*c*][1,2,5]thiadiazole. However, for acceptor groups with strong electron withdrawing capacity, almost no energy shift was observed between NTMB and PNT, and NSMB and PNS.

A typical synthesis for PBT-, PNT- and PNS-based Zn-LMOFs, HIAM-300*X* (HIAM = Hoffmann Institute of Advanced Materials; 30 = zinc; *X* = 1 for PBT, *X* = 2 for PNT and *X* = 3 for PNS) is as follows: a 5 mL vial containing 0.1 mmol Zn(NO_3_)_2_·6H_2_O, 0.1 mmol 1,4-dicarboxybenzene, 0.1 mmol designed linker, 1 mL DMF and 3 mL water was placed in a preheated oven at 120 °C for 3 days. After cooling down to room temperature, the corresponding single crystals were obtained.

The pillar-layered structure of HIAM-3001 has been reported to have a **pcu** topology and belongs to an orthorhombic crystal system with a *Pbca* space group.^19^ As shown in Fig. 3b, two equivalent Zn(ii) are bridged by four carboxylate groups from four BDC ligands to form a binuclear “paddle-wheel” Zn_2_(COO)_4_. These 6-c second building units (SBUs) are connected by the BDC ligands to give (4, 4) layers which are further extended by PBT ligands to form the 3D network with a **pcu** topology. Finally, two of these 3D networks generate a 2-fold interpenetrated framework.

HIAM-3002 and HIAM-3003 form a similar 2-fold interpenetrating **pcu** framework as HIAM-3001, while significant structural distortions are introduced by replacing the donor group from benzo[*c*][1,2,5]thiadiazole to naphtho[2,3-*c*][1,2,5]thiadiazole or naphtho[2,3-*c*][1,2,5]selenadiazole. As depicted in Fig. 3c and S3,† the SBUs are changed from the 6-c “paddle-wheel” (Zn_2_(*μ*_1,3_-COO)_4_N_2_) to the 6-c binuclear structure (Zn_2_(*μ*_1,3_-COO)_2_(COO)_2_N_4_). Two Zn(ii) atoms are bridged by two carboxylate groups, and the remaining coordination sites are occupied by four N atoms from two pairs of PNT (or PNS) ligands and two O atoms from two BDC ligands. Those binuclear SBUs are further connected by BDC ligands and PNT (or PNS) ligands to give the 3D **pcu** network with 2-fold interpenetration. Strong π–π interactions are found between the naphtho[2,3-*c*][1,2,5]thiadiazole rings in HIAM-3002 (centroid-to-centroid distance is 3.5317(3) Å), and naphtho[2,3-*c*][1,2,5]selenadiazole rings in HIAM-3003 (centroid-to-centroid distance is 3.5925(3) Å).

The PXRD patterns of the as-synthesized LMOFs exhibit excellent agreement with the simulated ones, indicating the high purity of the obtained bulk samples (Fig. 3d). The peak maxima of solid-state emission of HIAM-3001, HIAM-3002 and HIAM-3003 are 499 nm, 592 nm and 678 nm, respectively (Fig. 3e), with PLQYs of 2.8%, 0.6% and 1.0% under 365 nm excitation. The lower PLQY could be attributed to the fact that the π–π stacking is much stronger in pillar-layered structures, which will cause severe non-radiative decay. The gradual red-shift was also observed in the UV-vis absorption spectra from HIAM-3001 to HIAM-3003 (Fig. 3f). Compared with UiO-68 type MOFs, HIAM-3001, 3002 and 3003 show high stability in aqueous solutions after treatment at pH = 2 to 12 for one day, confirmed by the nearly identical PXRD patterns (Fig. S4 and S5†). HIAM-300*X* (*X* = 1–3) also exhibits high resistance to heat and stability up to 350 °C for HIAM-3001 and 400 °C for HIAM-3002 and HIAM-3003, respectively (Fig. S6†). The above results confirmed our hypothesis that changing the donor group to form a different metal–linker bond model will not only give rise to tunable emission behavior, but also contribute to structural diversity of the resultant LMOFs.

**Fig. 4:**
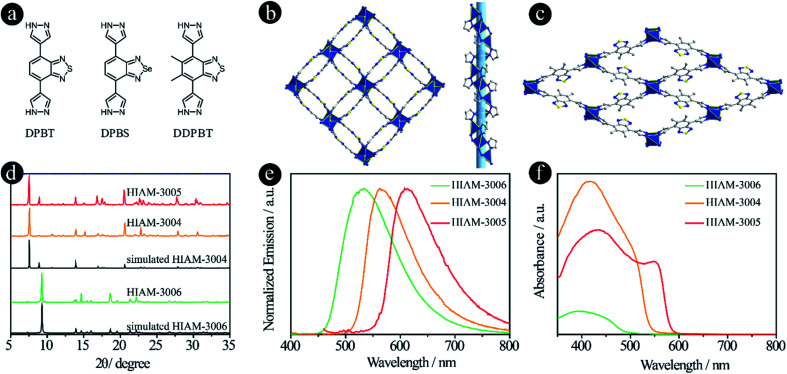
D–A–D type organic linkers with pyrazolate as the donor group. (a) The molecular structures of DPBT, DPBS and DDPBT; (b) the crystal structure of HIAM-3004 viewed along the *c* axis; (c) the crystal structure of HIAM-3006 viewed along the *a* axis; (d) the PXRD patterns of simulated and the as-synthesized HIAM-3004, HIAM-3005 and HIAM-3006; (e) the solid-state emission spectra and (f) the UV-vis absorption spectra of HIAM-3004, HIAM-3005 and HIAM-3006.

In recent years, pyrazolate-based MOFs have received considerable attention due to the pyrazolate–metal bond induced high stability and unique properties.^27–33^ Therefore, three pyrazolate-based linkers, 4,7-di(1*H*-pyrazol-4-yl)benzo[*c*][1,2,5]thiadiazole (DPBT), 4,7-di(1*H*-pyrazol-4-yl)benzo[*c*][1,2,5]selenadiazole (DPBS) and 5,6-dimethyl-4,7-di(1*H*-pyrazol-4-yl)benzo[*c*][1,2,5]thiadiazole (DDPBT), were designed and synthesized to study their effect on the emission properties of their corresponding LMOFs (Fig. 4a). Compared with MBTB (460 nm), BTMB (500 nm) and BSMB (530 nm), the emission peaks of DDPBT, DPBT and DPBS are 557 nm, 585 nm and 632 nm in DMF solution under 365 nm excitation (Fig. S7†). These results demonstrate that pyrazolate is a stronger electron donating group compared with carboxylic acid-based linkers adapted to the same acceptors, which can induce a strong bathochromic shift.

The synthesis conditions for pyrazolate-based HIAM-300*X* (*X* = 4 for DPBT, *X* = 5 for DPBS and *X* = 6 for DDPBT) are similar to those used to synthesize HIAM-3001 but without addition of 1,4-dicarboxybenzene. Single-crystal X-ray diffraction analysis reveals that HIAM-3004 crystallizes in the tetragonal crystal system with an *I*4_1_ space group (Fig. 4b and S8†). The Zn(ii) cation is fully coordinated in a tetrahedral geometry with four nitrogen atoms from four DPBT ligands. Each DPBT ligand is fully coordinated with four Zn(ii) cations, in which each pyrazolate group of the ligand connects two adjacent Zn(ii) cations. The alternative connection of Zn atoms and pyrazolate groups results in an infinite 4_1_ helical chain along the *c* axis (Fig. S9†). These screw chains are further extended by the DPBT ligand to give the 3D framework with 3D channels. Strong π–π interactions are found between the benzo[*c*][1,2,5]thiadiazole rings in HIAM-3004 (centroid-to-centroid distance is 3.5822(3) Å). An identical crystal structure was formed when DPBS was employed as the luminescent linker. It should be noted that a similar MOF to HIAM-3004 was reported for photocatalytic aerobic oxidation when we prepared our manuscript, which further demonstrates the promising applications of these MOFs.^34^

A totally different crystal structure was obtained when the acceptor group was changed from benzo[*c*][1,2,5]thiadiazole to 5,6-dimethylbenzo[*c*][1,2,5]thiadiazole for pyrazolate-based linkers. For HIAM-3006, a similar connection model to HIAM-3004 was observed where the Zn(ii) cation is fully coordinated in a tetrahedral geometry with four nitrogen atoms from four DDPBT ligands. Each DDPBT ligand is fully coordinated with four Zn(ii) cations, in which each pyrazolate group of the ligand connects two adjacent Zn(ii) cations to give a 1D Zn chain along the *a*-axis. The Zn chains are further extended by the DDPBT ligand to yield a 3D framework with 1D channels along the *a*-axis (Fig. 4c and S10†).

The PXRD patterns of the as-synthesized HIAM-3004 and HIAM-3005 show excellent agreement with the simulated HIAM-3004 pattern (Fig. 4d), indicating the phase purity and isoreticular nature of HIAM-3004 and HIAM-3005. HIAM-3006 also exhibits an essentially identical PXRD pattern to the simulated one. As shown in the emission spectra in Fig. 4e, compared with the emission peak maxima of UiO-68-MBTB (470 nm), UiO-68-BTMB (520 nm) and UiO-68-BSMB (545 nm), strong bathochromic shifts were recorded for HIAM-3006, HIAM-3004 and HIAM-3005 at 534 nm, 563 nm and 616 nm with the corresponding PLQYs of 8.5%, 0.6% and 28.9% under 365 nm excitation (Fig. 4e). A significant red-shift was also observed for the light absorption from HIAM-3006 to HIAM-3004 (Fig. 4f). HIAM-300*X* (*X* = 4–6) also exhibits high stability in aqueous solutions from pH 2 to 12 and thermal stability up to 500 °C (Fig. S11 and S12†).

By comparing the emission properties of UiO-68-BTMB (520 nm), HIAM-3001 (499 nm) and HIAM-3004 (563 nm), it is clear that introducing a pyridine group as the donor will induce a blue-shift, while a red-shift is observed when using pyrazolate as the donor. More importantly, with the tunable donor groups, abundant structural diversity can be realized, which might lead to new properties and applications.

### Tuning bridging units

It has been reported previously that different bridging groups show significant effects on the light absorption and emission behavior of D–A–D type compounds. For example, an emission shift from 610 nm, 622 nm to 647 nm was observed upon adding an ethynyl or a vinyl group between the donor and acceptor.^24,25^ Incorporating thiophene groups into fluorophores could also increase the light absorption and emission.^35–37^ In our previous work, we found that when the acceptor groups change from benzo[*c*][1,2,5]thiadiazole to naphtho[2,3-*c*][1,2,5]thiadiazole, the emission shifts from 520 nm to 585 nm.^15^ According to these results, we believe that organic linkers with a significant red-shift might be synthesized if these two features are combined in one structure. On the other hand, benzo[*c*][1,2,5]thiadiazole and naphtho[2,3-*c*][1,2,5]thiadiazole based carboxylic compounds have been utilized to prepare LMOFs with tunable emissions, and thus the biggest challenge is how to synthesize carboxylic compounds with a bathochromic shift in their emissions, which may provide the opportunity to obtain organic-linker-based NIR LMOFs.

**Fig. 5:**
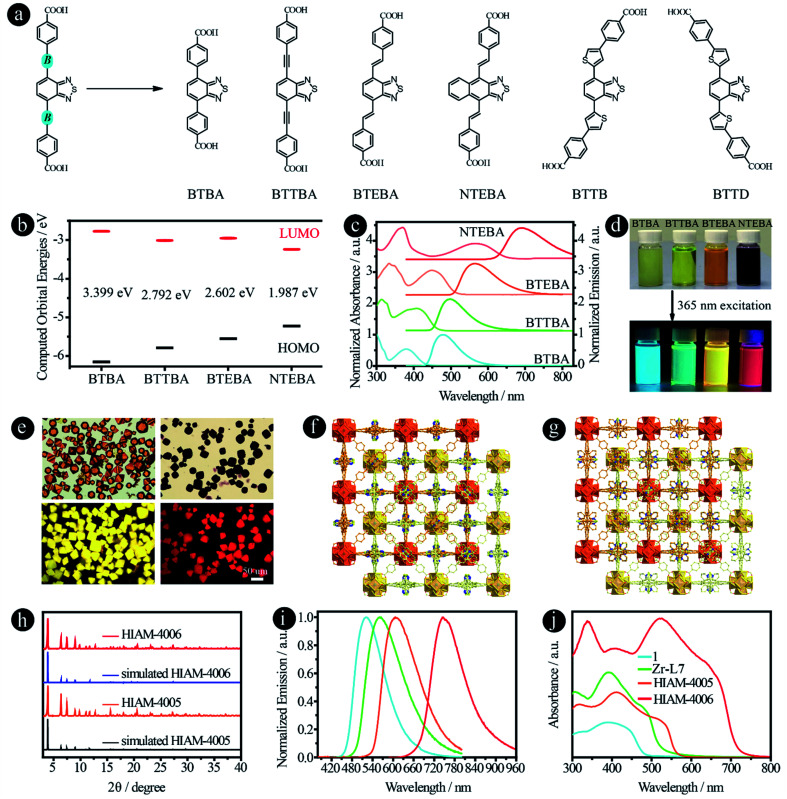
D–A–D type organic linkers with different bridging units between donor and acceptor groups. (a) The molecular structures of BTAB,^10^ BTTAB,^22^ BTEBA, NTEBA, BTTB and BTTD; (b) the calculated HOMO–LUMO energy levels, (c) normalized UV-vis absorption and emission spectra, and (d) the photographs of samples in DMF under daylight and 365 nm excitation of BTBA, BTTBA, BETBA, and NTEBA; (e) the single-crystal images under daylight (top) and 365 nm excitation (bottom), and (f) and (g) the crystal structures of HIAM-4005 and HIAM-4006; (h) the PXRD patterns of simulated and the as-synthesized HIAM-4005 and HIAM-4006; (i) normalized solid-state emission spectra and (j) UV-vis absorption spectra of 1, Zr-L7, HIAM-4005 and HIAM-4006.

To prove our hypothesis, six organic linkers were utilized in this section (Fig. 5a). Two of them have been reported, 4,4′-(benzo[*c*][1,2,5]thiadiazole-4,7-diyl)dibenzoic acid (BTBA)^10^ and 4,4′-(benzo[*c*][1,2,5]thiadiazole-4,7-diylbis(ethyne-2,1-diyl))dibenzoic acid (BTTBA).^22^ And the other four are newly synthesized linkers, namely 4,4′-(1*E*,1′*E*)-2,2′-(benzo[*c*][1,2,5]thiadiazole-4,7-diyl)bis(ethene-2,1-diyl)dibenzoic acid (BTEBA), 4,4′-(1*E*,1′*E*)-2,2′-(naphtho[2,3-*c*][1,2,5]thiadiazole-4,9-diyl)bis(ethene-2,1-diyl)dibenzoic acid (NTEBA), 4,4′-(5,5′-(benzo[*c*][1,2,5]thiadiazole-4,7-diyl)bis(thiophene-5,3-diyl))dibenzoic acid (BTTB) and 4,4′-(5,5′-(benzo[*c*][1,2,5]thiadiazole-4,7-diyl)bis(thiophene-5,2-diyl))dibenzoic acid (BTTD). BTBA, BTTBA and BTEBA were chosen to confirm the effect of bridging groups of ethynyl and vinyl. NTEBA was designed to achieve NIR emission. BTTB and BTTD were prepared to investigate the effect of thiophene and the substitution sites on the emission behaviors. The molecular orbitals of BTBA, BTTBA, BTEBA and NTEBA were calculated using density functional theory (DFT). As depicted in Fig. 5b, a significant increase in the highest occupied molecular orbital (HOMO) energies was observed along with a decrease in the lowest unoccupied molecular orbital (LUMO) energies when an ethynyl or a vinyl group was added. As a result, the HOMO–LUMO energy gap decreased from 3.399 eV to 2.792 eV and 2.602 eV for BTBA, BTTBA and BTEBA, respectively, indicating that the bridging groups indeed can be used to effectively tune the electronic structures. More importantly, a further decrease was obtained from 2.602 eV to 1.987 eV, when the acceptor group was changed from benzo[*c*][1,2,5]thiadiazole to naphtho[2,3-*c*][1,2,5]thiadiazole.

The UV-vis absorption and steady-state emission spectra of the four organic linkers in DMF solution were then measured and plotted in Fig. 5c. As expected, the emission energy varies from blue (BTBA) to NIR (NTEBA), which is consistent with the red-shifts of the corresponding absorption spectra. The maximum emission peaks appear at 480, 500, 565 and 690 nm for BTBA, BTTBA, BTEBA and NTEBA, respectively. The corresponding colors of the samples under daylight and 365 nm excitation are shown in Fig. 5d. A closer look at the molecular structures reveals an interesting structure-emission correlation. (i) Compared with BTBA, when a vinyl group is added between the donor and acceptor groups, an 85 nm bathochromic shift was observed for BTEBA. The remarkable effect of vinyl groups on the emission behavior could also be proven by comparison of the emission wavelength of NTEBA and the compound without vinyl groups, 4,4′-(naphtho[2,3-*c*][1,2,5]thiadiazole-4,9-diyl)dibenzoic acid (NTB), in which a bathochromic-shift of 100 nm was recorded after the addition of vinyl groups to NTB to form NTEBA. (ii) When acceptors were changed from benzo[*c*][1,2,5]thiadiazole to naphtho[2,3-*c*][1,2,5]thiadiazole, a 125 nm red-shift was realized. These results demonstrate that the combination of these two strategies together indeed offers a powerful approach to tune the emission properties and to achieve dicarboxylic acid-based NIR emissive organic linkers.

The single crystals of BTBA and BTTBA based Zr-MOFs (1 and Zr-L7) were synthesized according to literature procedures (Fig. S13†).^10,22^ A typical synthesis of BTEBA and NTEBA based Zr-MOFs, HIAM-400*X* (HIAM = Hoffmann Institute of Advanced Materials; 40 = zirconium; *X* = 5 for BETBA and *X* = 6 for NTEBA) is as follows: a 5 mL vial containing 54.0 mg l-proline and 63.0 μL was placed in a preheated oven at 100 °C for 30 minutes; after cooling down to room temperature, 3 mL DMF, ZrCl_4_ (22.0 mg, 0.094 mmol), and an organic linker (0.094 mmol) were added to the vial, which was placed in a preheated oven at 120 °C for 48 hours. Orange (HIAM-4005) and dark red (HIAM-4006) octagon-shaped single crystals were obtained (Fig. 5e). Single-crystal X-ray diffraction analysis indicates that HIAM-4005 and HIAM-4006 adopt the typical cubic structure and crystallize in the space group *Fd*3̄*m*. The structure consists of two sets of independent and mutually interpenetrating UiO-type frameworks (Fig. 5f and g). Therefore, the structure and connectivity of the SBUs in HIAM-4006 are the same as in UiO-type MOFs. The two new LMOFs show nearly identical powder X-ray diffraction (PXRD) patterns to the simulated one (Fig. 5h), indicating that these LMOFs made of linkers with different acceptor groups belong to the same isoreticular series.

The solid-state photoluminescence and UV-vis absorption spectra were measured. As shown in Fig. 5i, the emission maxima at 522 nm, 560 nm, 607 nm and 747 nm were recorded for 1, Zr-L7, HIAM-4005 and HIAM-4006 with the corresponding PLQYs of 7.8%, 5.6%, 8.8% and 0.5% under 365 nm excitation, respectively. The NIR emissive LMOF (HIAM-4006) was obtained by the combination of an acceptor with strong electron withdrawing capacity and an optimized bridging unit. A gradual red-shift was also observed in the absorption spectra from 1 to HIAM-4006, which is consistent with their corresponding emission energies (Fig. 5j). The lowest energy absorption edge is close to 750 nm, which may be suitable for applications in photocatalytic related areas, such as photocatalytic hydrogen generation and carbon dioxide reduction.

In addition to ethynyl and vinyl bridging units, thiophene was also utilized to link the donor and acceptor groups. Compared with BTBA (480 nm), BTTBA (500 nm) and BTETA (565 nm), the maximum emission peaks of BTTB and BTTD are 553 nm and 638 nm in DMF solution under 365 nm excitation, respectively (Fig. S14†). This result indicates that (i) thiophene is a useful bridging unit to achieve the bathochromic-shift; and (ii) the substitution position has a significant effect on the emission properties of the resultant compounds. However, it is very difficult to obtain the crystal structure of the corresponding LMOFs, which might be attributed to the distorted molecular structures.

**Fig. 6:**
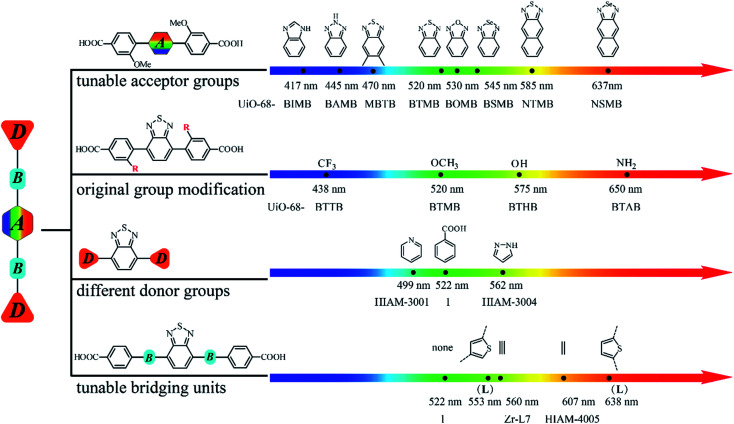
A systematic approach to building an emission library of LMOFs using tunable D–A–D type organic linkers.

Based on the aforementioned results, it is clear that the emission properties of D–A–D type organic linker-based LMOFs can be systematically tuned by changing the acceptor groups, modification of the original donor groups, using different donor groups and choosing various bridging units. Therefore, an emission library can thus be built by applying different strategies described in this work (Fig. 6).

## Conclusion

In conclusion, four strategies have been used to methodically tune the emission behaviors of D–A–D type organic linker-based LMOFs, from which a large emission library can be built in a systematic manner. By precisely controlling the acceptor groups, donor groups and the bridging units, emissions of the resultant LMOFs covering the entire visible light spectrum as well as the NIR region can be achieved with abundant structural diversity. A large number of organic linkers can be designed and synthesized with varying emission behaviors from deep blue to the NIR range by gradually decreasing the electron density of acceptor groups or increasing the electron density of donor groups. This work may not only serve as a toolbox to facilitate the development of design principles for the rational design of organic linkers and customized synthesis of target-specific LMOFs but also provide a useful platform to explore NIR emissive LMOFs for biosensing and bioimaging applications.

## Data availability

All crystallographic data have been deposited in the CSD. No other data is present.

## Author contributions

X.-Y. L. conceived the idea and designed the experiment; H.-L. Xia, S. Wu and D. Ren worked on the synthesis and the characterization studies of all materials. K. Zhou and K. Xing carried out the analysis of the single crystal structures. X. Wang and J. Guo did the density functional theory calculations. X.-Y. L. and J. Li wrote the paper with help from all authors. H.-L. Xia and K. Zhou contributed equally to this work.

## Conflicts of interest

The authors declare no competing financial interests.

## Supplementary Material

SC-013-D2SC02267B-s001

SC-013-D2SC02267B-s002
