# Supplementary material for: Building an emission library of donor–acceptor–donor type linker-based luminescent metal–organic frameworks
Source: Chem Sci. 2022 Jun 17;13(27):8036–44. doi: 10.1039/d2sc02267b (PMC9278489; doi:10.1039/d2sc02267b)
Supplement: SC-013-D2SC02267B-s001 [file SC-013-D2SC02267B-s001.pdf]

Electronic Supplementary Material (ESI) for Chemical Science.
This journal is © The Royal Society of Chemistry 2022

Supporting Information

Building an Emission Library of Donor-Acceptor-Donor Type Linker-Based Luminescent Metal-Organic Frameworks

Hai-Lun Xia,[†,+] Kang Zhou,[†,+] Shenjie Wu,[†] Daming Ren,[†] Kai Xing,[‡] Jiandong Guo,[†] Xiaotai Wang,[§] Xiao-Yuan Liu,[*,†] and Jing Li[¶,†]

[†] Hoffmann Institute of Advanced Materials, Shenzhen Polytechnic, 7098 Liuxian Blvd, Nanshan District, Shenzhen, 518055, P. R. China

[‡] Department of Chemistry, College of Basic Medicine, Third Military Medical University (Army Medical University), Chongqing, 400038, P. R. China

[§] Department of Chemistry, University of Colorado Denver, Campus Box 194, P. O. Box 173364, Denver, Colorado 80217-3364, United States

[¶] Department of Chemistry and Chemical Biology, Rutgers University, 123 Bevier Road, Piscataway, New Jersey 08854, United States

[+] These authors contributed equally to this work.

Chemicals

4,9-dibromonaphtho[2,3-*c*][1,2,5]thiadiazole was purchased from Jilin Chinese Academy of Science-Yanshen Technology Co., Ltd. 4,7-dibromo-1H-benzo[d]imidazole, 4,7-dibromobenzo[c][1,2,5]oxadiazole, methyl-3-amino-4-iodobenzoate, methyl-3-hydroxy-4-iodobenzoate, methyl-4-bromo-3-(trifluoromethyl)benzoate, 4,7-dibromobenzo[c][1,2,5]thiadiazole, 4-pyridinylboronic acid, 2,5-dibromothiophene, 2,4-dibromothiophene, 4-(ethoxycarbonyl)phenylboronic acid, 1,4-dicarboxybenzene (BDC) and 4,7-bis(4,4,5,5-tetramethyl-1,3,2-dioxaborolan-2-yl)benzo[c][1,2,5]thiadiazole were purchased from Shanghai Aladdin Biochemical Technology Co., Ltd. Methyl 2-methoxy-4-(methoxycarbonyl)phenylboronic acid pinacol ester, 4,7-dibromo-5,6-dimethylbenzo[c][1,2,5]thiadiazole (2a)[1], 4,7-dibromobenzo[c][1,2,5]selenadiazole (2c)[2], 4,9-dibromonaphtho[2,3-c][1,2,5]selenadiazole (2e)[3] and 4-(4,4,5,5-tetramethyl-1,3,2-dioxaborolan-2-yl)-1-trityl-1H-pyrazole were synthesized according to the reported methods. All the other chemicals were obtained from the chemical supplies and used without further purification.

Characterization

Nuclear magnetic resonance (NMR) data was collected using 400 MHz JEOL JNM-ECZ400S. Powder X-ray diffraction (PXRD) patterns were recorded using Bruker D8 Advance X-ray diffractometer with Cu Kα radiation. Single crystal X-ray diffraction data were collected at 100 K on a Bruker D8 Venture diffractometer. The photoluminescent spectra were measured on FLS1000 spectrofluorometer (Edinburgh Instruments). The UV-vis spectra were recorded on Shimadzu UV-3600 spectrophotometer. The quantum yield was measured using C9920-03

absolute quantum yield measurement system (Hamamatsu Photonics) with a 150 W xenon monochromatic light source and 3.3 inch integrating sphere. The TGA data was collected using TGA 550 (TA Instruments) analyzer and the samples were heated from room temperature to 600°C at a ramp rate of 10°C / min.

Density functional theory calculations

Structures of BTBA, BTTBA, BTEBA and NTEBA were optimized and characterized by frequency calculations to be energy minima (zero imaginary frequencies) at the B3LYP[4]/6-31g(d,p) level of density functional theory (DFT). The HOMO–LUMO gaps were then calculated from the output files. All calculations were performed using Gaussian 09.[5]

Linker synthesis

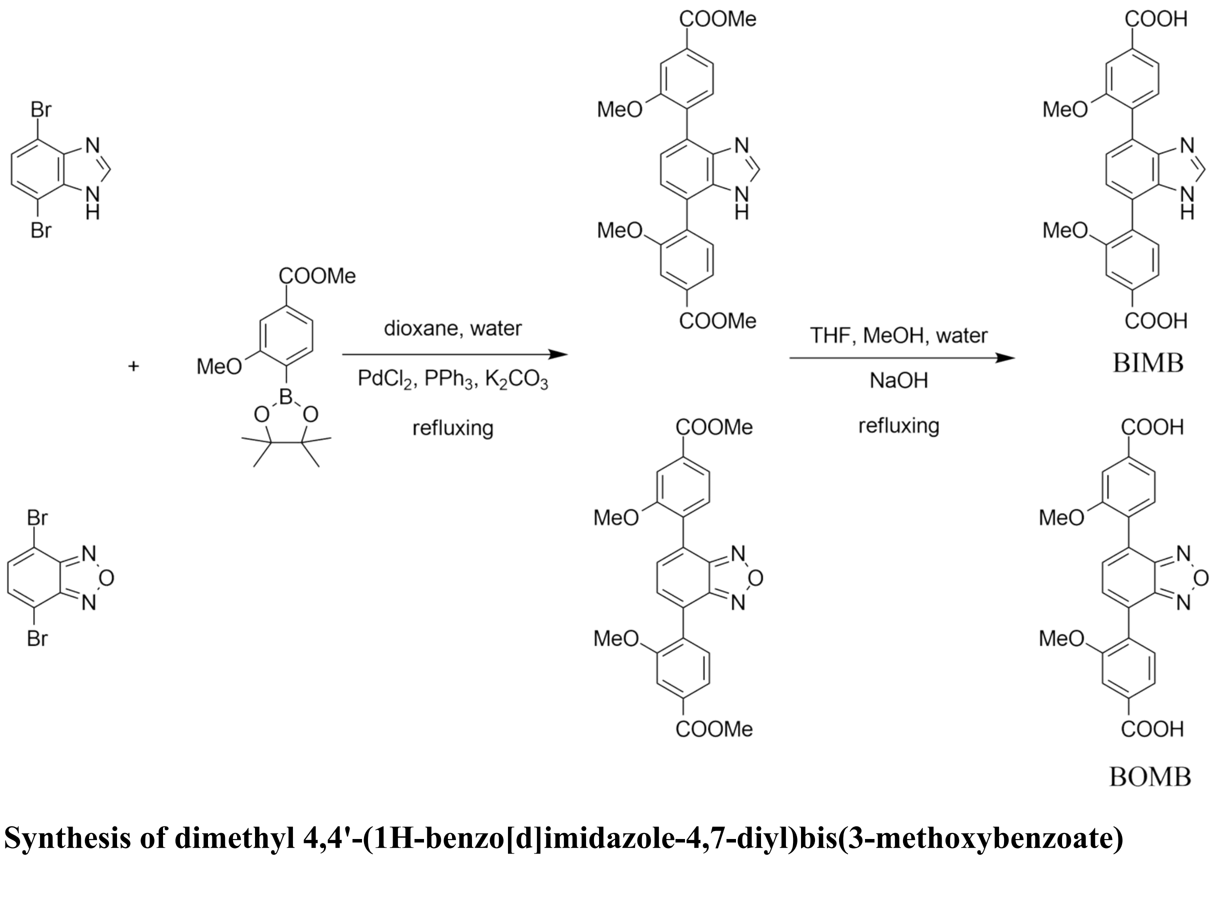

Synthesis of dimethyl 4,4'-(1H-benzo[d]imidazole-4,7-diyl)bis(3-methoxybenzoate)

Methyl 2-methoxy-4-(methoxycarbonyl)phenylboronic acid pinacol ester (10.0 mmol, 2.92 g), 4,7-dibromo-1H-benzo[d]imidazole (4.0 mmol, 1.10 g), $PdCl_2$ (0.4 mmol, 70.0 mg), PPh_3 (0.8

mmol, 0.21 g) and K_2CO_3 (16.0 mmol, 2.20 g) were added in a solution containing 120 mL dioxane and 30 mL water. The reaction solution was degassed four times. Then the mixture was heated to reflux at 105 °C for 24 h under nitrogen atmosphere. After cooling down to room temperature, the solvent was removed under reduced pressure and 100 mL ID water was added. The crude product was obtained after filtration and washed using ID water, which was directly used for next step without further purification (yield: 76.2%). ^{1}H NMR (400 MHz, $CDCl_3$) δ ppm 8.05 (1H), 7.78 (2H), 7.73 (2H), 7.64 (2H), 7.46 (2H), 3.95 (6H), 3.90 (6H).

Synthesis of 4,4'-(1H-benzo[d]imidazole-4,7-diyl)bis(3-methoxybenzoic acid) (BIMB)

Dimethyl 4,4'-(1H-benzo[d]imidazole-4,7-diyl)bis(3-methoxybenzoate) (2.0 mmol, 0.89 g) was added to a solution containing 25 mL CH_3OH, 50 mL THF and 25 mL water with 2.5 g NaOH. The mixture was heated to reflux at 80 °C overnight. After cooling down to room temperature, the organic solvent was removed under reduced pressure and the resulted aqueous was filtered. Then the filtrate was neutralized using 2M HCl to obtain the precipitate, which was filtered to offer the final product 4,4'-(1H-benzo[d]imidazole-4,7-diyl)bis(3-methoxybenzoic acid) BIMB as a white solid (0.79 g, 94.5%). ^{1}H NMR (400 MHz, DMSO-d_6) δ ppm 9.64 (1H), 7.66 (4H), 7.57 (4H), 3.81 (6H). ^{13}C NMR (101 MHz, DMSO-d_6) 167.548, 156.830, 141.431, 133.047, 131.581, 129.596, 128.769, 127.093, 125.144, 122.269, 112.092, 55.668.

Dimethyl 4,4'-(benzo[c][1,2,5]oxadiazole-4,7-diyl)bis(3-methoxybenzoate) was synthesized using the similar method as dimethyl 4,4'-(1H-benzo[d]imidazole-4,7-diyl)bis(3-methoxybenzoate) (yield: 88.3%). ^{1}H NMR (400 MHz, $CDCl_3$) δ ppm 8.08 (4H), 7.73 (2H), 7.66 (2H), 3.95 (6H), 3.91 (6H).

4,4'-(benzo[c][1,2,5]oxadiazole-4,7-diyl)bis(3-methoxybenzoic acid) (BOMB) was synthesized using the similar method as BIMB (yellow green solid, yield: 96.4%). ^{1}H NMR (400 MHz, DMSO-d_6) δ ppm 7.72 (2H), 7.65 (6H), 3.80 (6H). ^{13}C NMR (101 MHz, DMSO-d_6) 167.418, 157.309, 149.217, 133.295, 132.954, 131.654, 128.647, 125.925, 122.223, 112.553, 56.353.

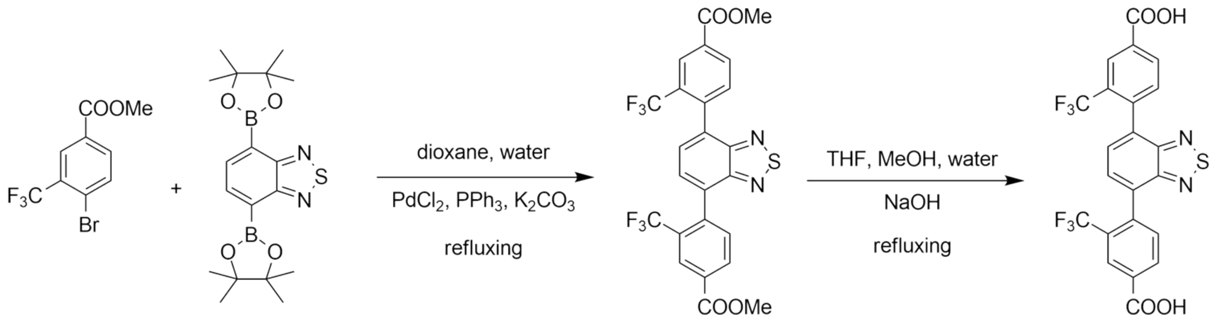

BTTB

Synthesis of dimethyl 4,4'-(benzo[c][1,2,5]thiadiazole-4,7-diyl)bis(3-(trifluoromethyl)benzoate)

Methyl-4-bromo-3-(trifluoromethyl)benzoate (8.2 mmol, 2.32 g), 4,7-Bis(4,4,5,5-tetramethyl-1,3,2-dioxaborolan-2-yl)-2,1,3-benzothiadiazole (4.0 mmol, 1.55 g), $PdCl_2$ (0.4 mmol, 70 mg), PPh_3 (0.8 mmol, 0.21 g) and K_2CO_3 (16 mmol, 2.2 g) were added in a solution containing 120 mL dioxane and 30 mL water. The reaction solution was degassed four times. Then the mixture was heated to reflux at 105 °C for 6 h under nitrogen atmosphere. After cooling down to room temperature, the solvent was removed under reduced pressure and 100 mL water was added. The mixture was extracted using dichloromethane for three times. The organic phase was combined and dried over Na_2SO_4. The product was obtained after purifying by silica gel column chromatography as a light yellow solid (1.45 g, yield: 67.1%). ^{1}H NMR (400 MHz, $CDCl_3$) δ ppm 8.47 (2H), 8.35 (2H), 7.64 (4H), 3.92 (6H).

Synthesis of 4,4'-(benzo[c][1,2,5]thiadiazole-4,7-diyl)bis(3-(trifluoromethyl)benzoic acid)

(BTTB)

Dimethyl 4,4'-(benzo[c][1,2,5]thiadiazole-4,7-diyl)bis(3-aminobenzoate) (2.0 mmol, 1.08 g) was added to a solution containing 50 mL THF, 25 mL CH_3OH and 25 mL water with 2.0 g NaOH. The mixture solution was heated to reflux at 80 °C overnight. After cooling down to room temperature, the mixture was filtrated. The filtrate was removed under reduced pressure and the resulted aqueous was filtered again. Then the filtrate was neutralized using 2M HCl to obtain the precipitate, which was filtered to offer the final product 4,4'-(benzo[c][1,2,5]thiadiazole-4,7-diyl)bis(3-(trifluoromethyl)benzoic acid) (BTTB) as a light yellow solid (0.98 g, 95.7%). ^{1}H NMR (400 MHz, DMSO-d_6) δ ppm 8.35 (2H), 8.30 (2H), 7.76 (4H). ^{13}C NMR (101 MHz, DMSO-d_6) 166.387, 153.540, 140.173, 133.900, 133.257, 132.064, 131.590, 129.482, 128.976, 128.676, 127.256, 125.408, 122.682.

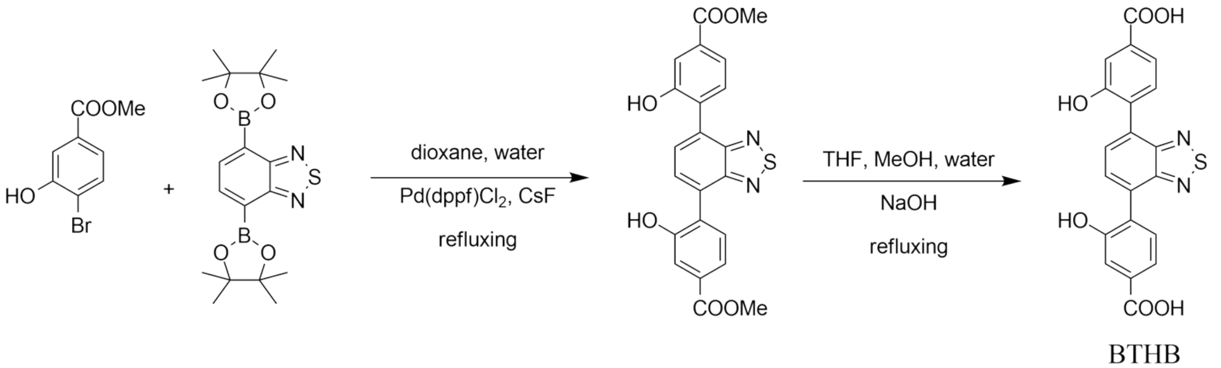

BTHB

Synthesis of dimethyl 4,4'-(benzo[c][1,2,5]thiadiazole-4,7-diyl)bis(3-hydroxybenzoate)

Methyl-3-hydroxy-4-iodobenzoate (8.2 mmol, 2.28 g), 4,7-Bis(4,4,5,5-tetramethyl-1,3,2-dioxaborolan-2-yl)-2,1,3-benzothiadiazole (4.0 mmol, 1.55 g), $PdCl_2$(dppf) (0.4 mmol, 0.29 g) and CsF (24 mmol, 3.64 g) were added in a solution containing 80 mL dioxane and 40 mL water. The reaction solution was degassed four times. Then the mixture was heated to reflux at 105 °C for 6 h under nitrogen atmosphere. After cooling down to room temperature, the mixture was filtrated. DI water was added to the filtrate to get the solid product. The crude product was

obtained after filtration and washed using DI water, which was directly used for next step without further purification (yellow solid, 1.65 g, 94.6%). ^{1}H NMR (400 MHz, DMSO-d_6) δ ppm 10.00 (2H), 7.82 (2H), 7.65-7.56 (4H), 7.55-7.46 (2H), 3.85 (6H).

Synthesis of 4,4'-(benzo[c][1,2,5]thiadiazole-4,7-diyl)bis(3-hydroxybenzoic acid) (BTHB)

Dimethyl 4,4'-(benzo[c][1,2,5]thiadiazole-4,7-diyl)bis(3-hydroxybenzoate) (3.0 mmol, 1.31 g) was added to a solution containing 100 mL THF, 50 mL CH_3OH and 50 mL water with 4.0 g NaOH. The mixture solution was heated to reflux at 80 °C overnight. After cooling down to room temperature, the mixture was filtrated. The filtrate was removed under reduced pressure and the resulted aqueous was filtered again. Then the filtrate was neutralized using 2M HCl to obtain the precipitate, which was filtered to offer the final product 4,4'-(benzo[c][1,2,5]thiadiazole-4,7-diyl)bis(3-hydroxybenzoic acid) (BTHB) as an orange solid (1.21 g, 98.1%). ^{1}H NMR (400 MHz, DMSO-d_6) δ ppm 9.99 (2H), 7.81 (2H), 7.58 (4H), 7.49 (2H). ^{13}C NMR (101 MHz, DMSO-d_6) 167.683, 155.498, 153.791, 132.312, 130.248, 129.139, 120.197, 117.004.

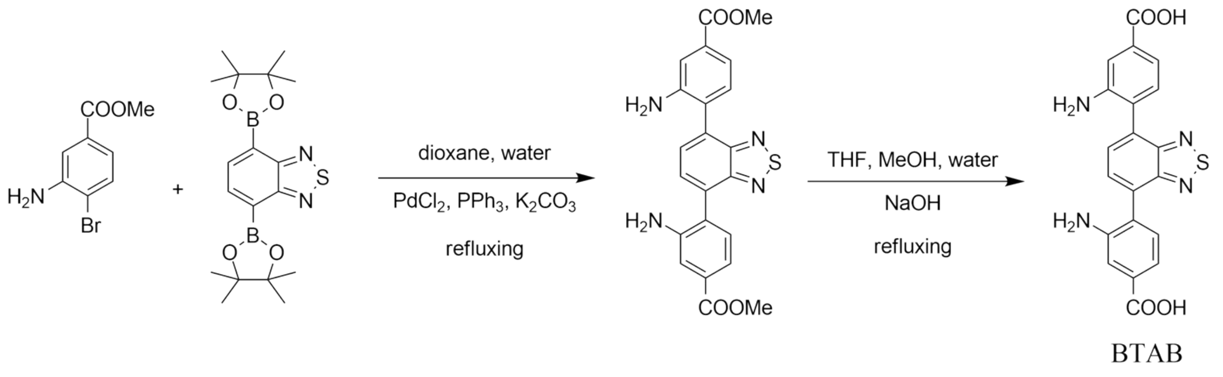

Synthesis of dimethyl 4,4'-(benzo[c][1,2,5]thiadiazole-4,7-diyl)bis(3-aminobenzoate)

Methyl-3-amino-4-iodobenzoate (8.2 mmol, 2.27 g), 4,7-Bis(4,4,5,5-tetramethyl-1,3,2-dioxaborolan-2-yl)-2,1,3-benzothiadiazole (4.0 mmol, 1.55 g), $PdCl_2$ (0.4 mmol, 70 mg), PPh_3 (0.8 mmol, 0.21 g) and K_2CO_3 (16 mmol, 2.2 g) were added in a solution containing 120 mL

dioxane and 30 mL water. The reaction solution was degassed four times. Then the mixture was heated to reflux at 105 °C for 6 h under nitrogen atmosphere. After cooling down to room temperature, the mixture was filtrated. DI water was added to the filtrate to get the solid product. The crude product was obtained after filtration and washed using DI water, which was directly used for next step without further purification (orange solid, 1.34 g, 71.9%). ^{1}H NMR (400 MHz, $CDCl_3$) δ ppm 7.78 (2H), 7.60-7.56 (4H), 7.39 (2H), 3.94 (6H).

Synthesis of 4,4'-(benzo[c][1,2,5]thiadiazole-4,7-diyl)bis(3-aminobenzoic acid) (BTAB)

Dimethyl 4,4'-(benzo[c][1,2,5]thiadiazole-4,7-diyl)bis(3-aminobenzoate) (3.0 mmol, 1.30 g) was added to a solution containing 100 mL THF, 50 mL CH_3OH and 50 mL water with 4.0 g NaOH. The mixture solution was heated to reflux at 80 °C overnight. After cooling down to room temperature, the mixture was filtrated. The filtrate was removed under reduced pressure and the resulted aqueous was filtered again. Then the filtrate was neutralized using 2M HCl to obtain the precipitate, which was filtered to offer the final product 4,4'-(benzo[c][1,2,5]thiadiazole-4,7-diyl)bis(3-aminobenzoic acid) (BTAB) as a brown solid (1.18 g, 96.9%). ^{1}H NMR (400 MHz, DMSO-d_6) δ ppm 7.72 (2H), 7.51 (2H), 7.35 (4H). ^{13}C NMR (101 MHz, DMSO-d_6) 167.594, 154.149, 141.126, 132.071, 131.911, 131.126, 130.721, 130.140, 121.690, 119.849.

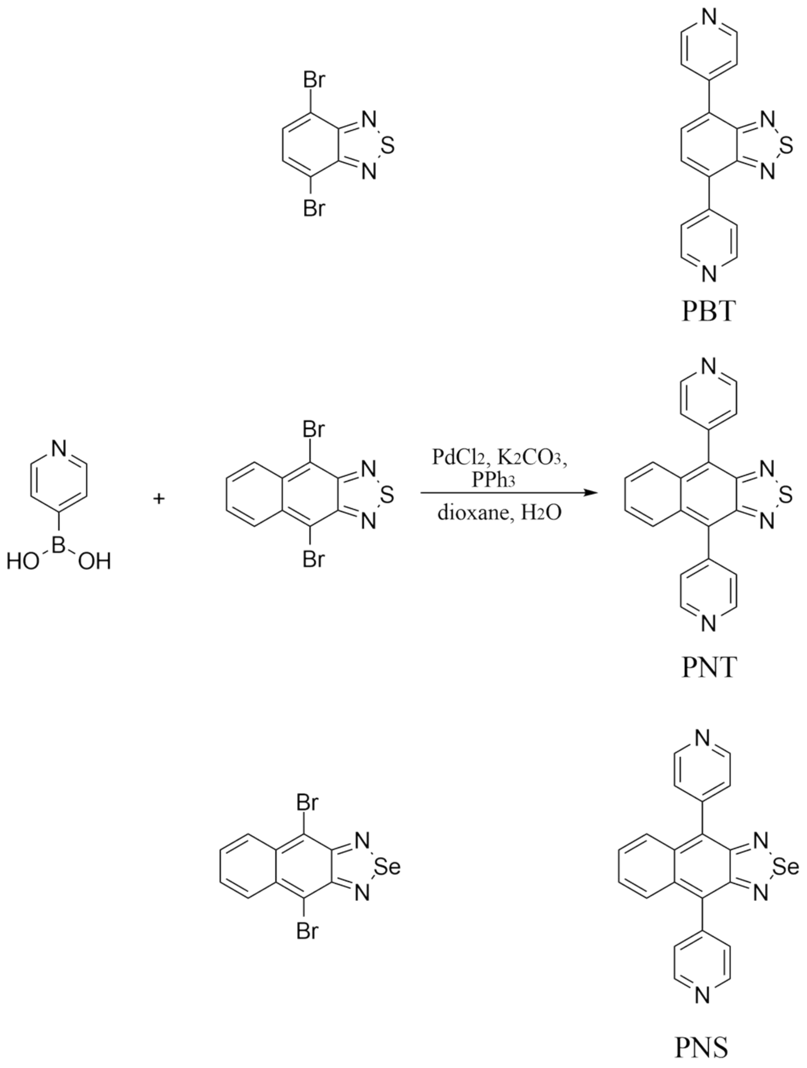

Synthesis of 4,7-di(pyridine-4-yl)benzo[c][1,2,5]thiadiazole (PBT)

4,7-dibromobenzo[c][1,2,5]thiadiazole (10.0 mmol, 2.93 g), 4-pyridinylboronic acid (25.0 mmol, 3.07 g), $PdCl_2$ (1.0 mmol, 0.17 g), PPh_3 (2.0 mmol, 0.52 g) and K_2CO_3 (40.0 mmol, 5.53 g) were added into a flask containing 120 mL 1,4-dioxane and 30 mL H_2O. After degassed four times, the mixture was heated and refluxed under nitrogen for 24 hours. After cooling to room temperature, the solvent was removed by rotary evaporator and the crude product was filtered and washed with water several times. The pure PBT was obtained as a pale yellow solid by flash column chromatography (2.23 g, 90.9%). ^1H NMR (400 MHz, $CDCl_3$) δ ppm 8.81 (4H), 7.94 (4H), 7.93 (2H). ^{13}C NMR (101 MHz, $CDCl_3$) 153.381, 150.208, 144.200, 132.028, 128.614, 123.686.

Synthesis of 4,9-di(pyridine-4-yl)naphtho[2,3-c][1,2,5]thiadiazole (PNT)

PNT was synthesized using the same route as PBT. Pure PNT was obtained as orange solid by flash column chromatography (3.24 g, 95.3%). ^{1}H NMR (400 MHz, $CDCl_3$) δ ppm 8.91 (4H), 7.95 (2H), 7.63 (4H), 7.46 (2H). ^{13}C NMR (101 MHz, $CDCl_3$) 150.653, 150.141, 144.396, 131.516, 128.221, 127.532, 126.483, 126.196, 121.240.

Synthesis of 4,9-di(pyridine-4-yl)naphtho[2,3-c][1,2,5]selenadiazole (PNS)

PNS was synthesized using the same route as PBT. Pure PNS was obtained as red solid by flash column chromatography (3.50 g, 90.3%). ^{1}H NMR (400 MHz, DMSO-d_6) δ ppm 8.78 (4H), 7.61 (6H), 7.35 (2H). ^{13}C NMR (101 MHz, $CDCl_3$) 156.794, 150.118, 144.970, 131.838, 129.033, 127.558, 126.636, 126.203.

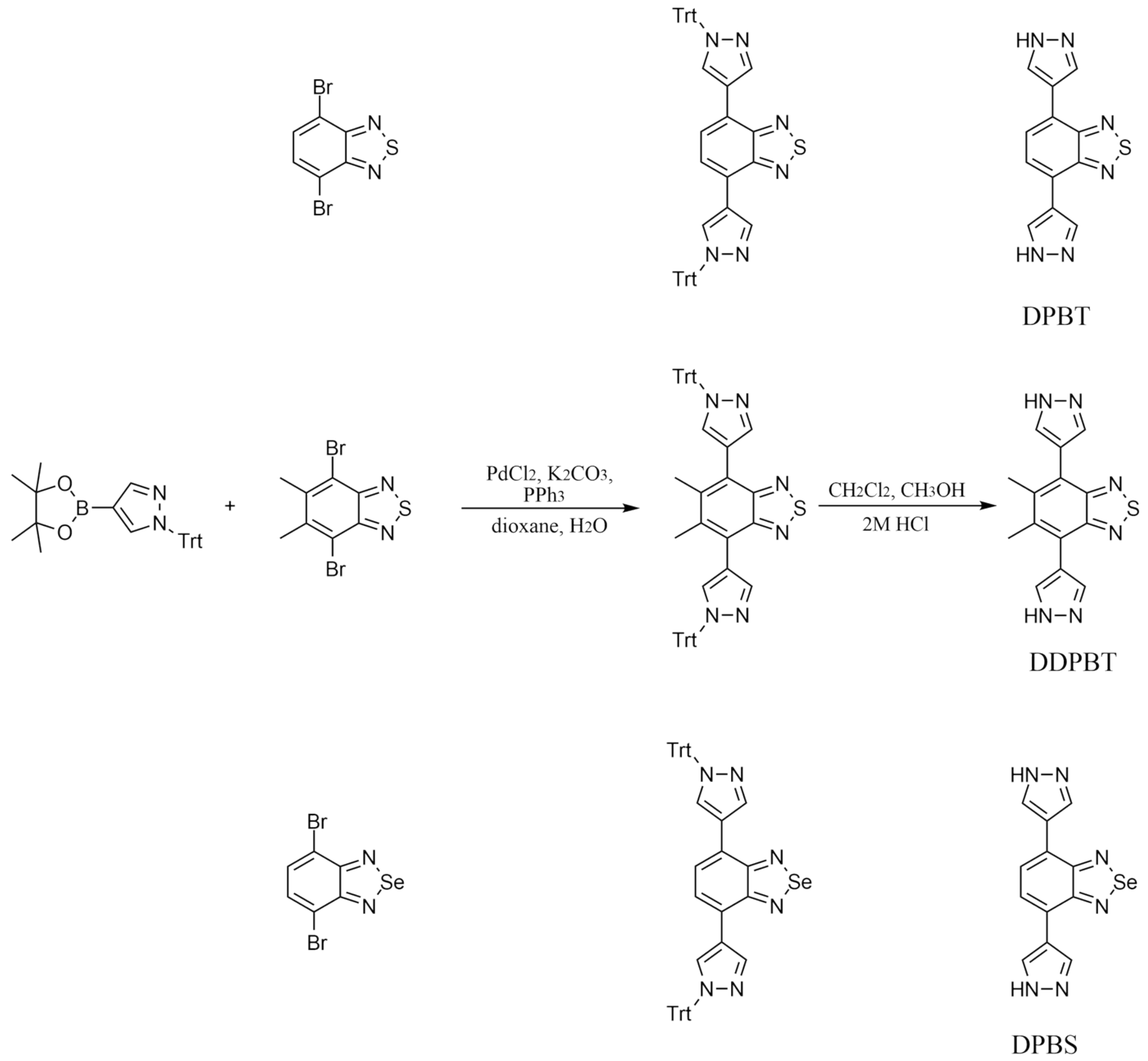

Synthesis of 4,7-bis(1-trityl-1H-pyrazol-4-yl)benzo[c][1,2,5]thiadiazole

4-(4,4,5,5-tetramethyl-1,3,2-dioxaborolan-2-yl)-1-trityl-1H-pyrazole (12.0 mmol, 5.23 g), 4,7-dibromo-2,1,3-benzothiadiazole (4.0 mmol, 1.17 g), $PdCl_2$ (0.8 mmol, 0.14 g), PPh_3 (0.80 mmol, 0.21 g) and K_2CO_3 (16.0 mmol, 2.20 g) were added into 120 mL 1,4-dioxane and 30 mL H_2O. After degassed four times, the mixture solution was heated and refluxed under nitrogen for 16 hours. After cooling to room temperature, the solvent was removed by rotary evaporator and the crude product was filtered and washed with water several times. 4,7-bis(1-trityl-1H-pyrazol-4-yl)benzo[c][1,2,5]thiadiazole was obtained as an orange solid (2.84 g, 94.5%). ^{1}H

NMR (400 MHz, $CDCl_3$) δ ppm 8.43 (2H), 8.36 (2H), 7.67 (2H), 7.35-7.31 (18H), 7.23 (12H).

Synthesis of 5,6-dimethyl-4,7-bis(1-trityl-1H-pyrazol-4-yl)benzo[c][1,2,5]thiadiazole

Compound 5,6-dimethyl-4,7-bis(1-trityl-1H-pyrazol-4-yl)benzo[c][1,2,5]thiadiazole was synthesized using the same route as 4,7-bis(1-trityl-1H-pyrazol-4-yl)benzo[c][1,2,5]thiadiazole. 5,6-dimethyl-4,7-bis(1-trityl-1H-pyrazol-4-yl)benzo[c][1,2,5]thiadiazole was obtained as light green solid (2.52 g, 80.8%). ^{1}H NMR (400 MHz, $CDCl_3$) δ ppm 7.95 (2H), 7.72 (2H), 7.32-7.27 (30H), 2.43 (6H).

Synthesis of 4,7-bis(1-trityl-1H-pyrazol-4-yl)benzo[c][1,2,5]selenadiazole

Compound 4,7-bis(1-trityl-1H-pyrazol-4-yl)benzo[c][1,2,5]selenadiazole was synthesized using the same route as 4,7-bis(1-trityl-1H-pyrazol-4-yl)benzo[c][1,2,5]thiadiazole. 4,7-bis(1-trityl-1H-pyrazol-4-yl)benzo[c][1,2,5]selenadiazole was obtained as red solid (2.95 g, 92.2%). ^{1}H NMR (400 MHz, DMSO-d_6) δ ppm 8.41 (2H), 8.33 (2H), 7.55 (2H), 7.34-7.31 (18H), 7.23 (12H).

Synthesis of 4,7-di(1H-pyrazol-4-yl)benzo[c][1,2,5]thiadiazole (DPBT)

Compound 1 (4.0 mmol, 3.01 g) was added into a mixture solution containing 60 mL DCM, 60 mL methanol and 40 mL 2M HCl. The mixture was heated 45°C for 6 hours. After cooling to room temperature, the solvent was removed under reduced pressure. The orange red solid was filtered and washed with water for several times (0.84 g, 78.5%). ^{1}H NMR (400 MHz, DMSO-d_6) δ ppm 8.50 (4H), 7.96 (2H). ^{13}C NMR (101 MHz, DMSO-d_6) 153.150, 133.254, 125.502, 123.605, 117.600.

Synthesis of 5,6-dimethyl-4,7-di(1H-pyrazol-4-yl)benzo[c][1,2,5]thiadiazole (DDPBT)

DDPBT was synthesized using the same route as DPBT. DDPBT was obtained as green solid

(0.93 g, 78.5%). ^{1}H NMR (400 MHz, DMSO-d$_6$) δ ppm 7.94 (4H), 2.45 (6H). ^{13}C NMR (101 MHz, DMSO-d_6) 153.406, 138.373, 135.116, 121.683, 116.622, 19.265.

Synthesis of 4,7-di(1H-pyrazol-4-yl)benzo[c][1,2,5]selenadiazole (DPBS)

DPBS was synthesized using the same route as DPBT. DPBS was obtained as red orange solid (1.04 g, 82.3%). ^{1}H NMR (400 MHz, DMSO-d$_6$) δ ppm 8.50 (4H), 7.97 (2H). ^{13}C NMR (101 MHz, DMSO-d_6) 158.771, 133.276, 125.097, 118.279.

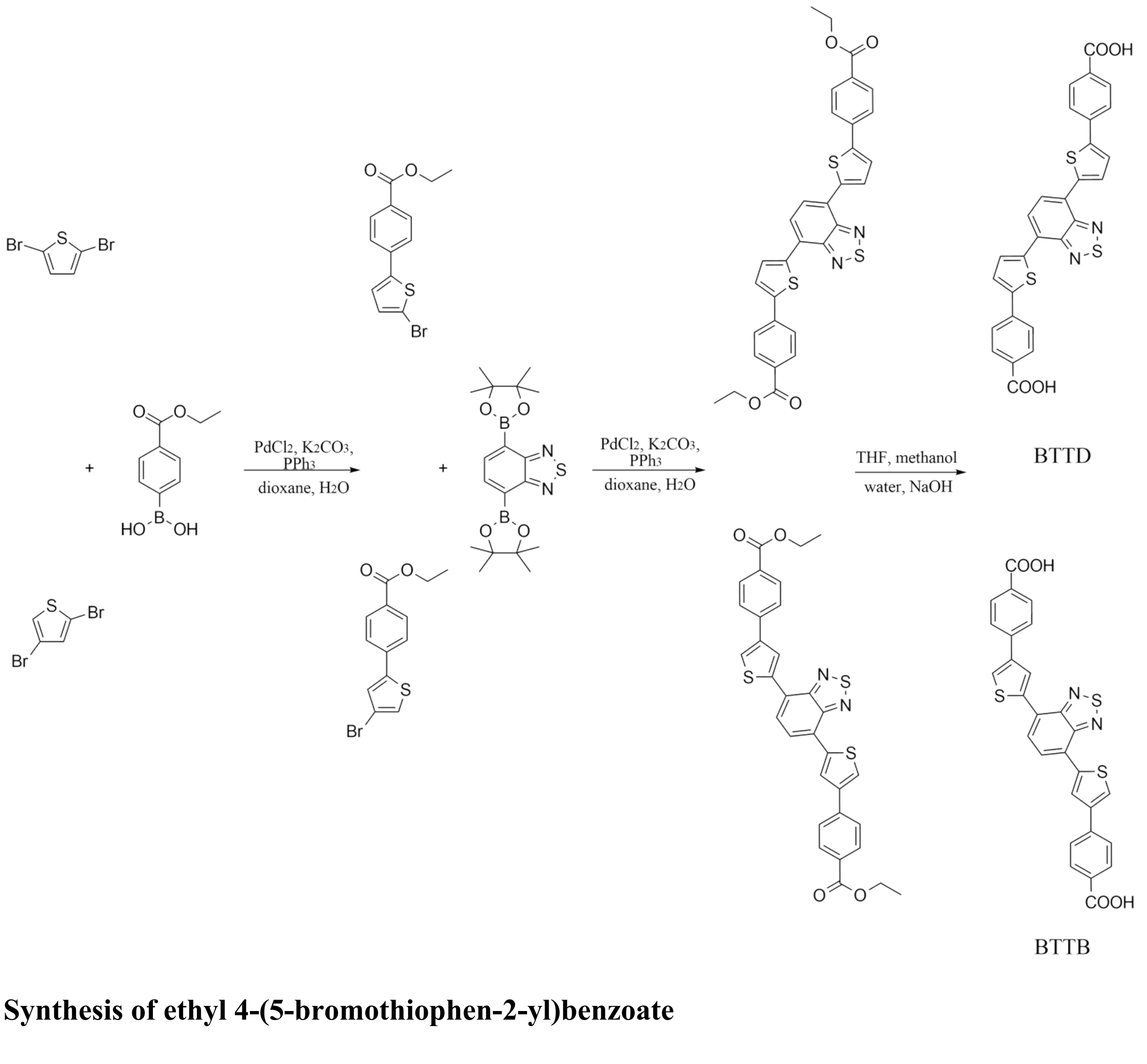

Synthesis of ethyl 4-(5-bromothiophen-2-yl)benzoate

2,5-dibromothiophene (30.0 mmol, 7.26 g), 4-(ethoxycarbonyl)phenylboronic acid (20.0 mmol, 3.88 g), $PdCl_2$ (0.8 mmol, 0.14 g), PPh_3 (1.60 mmol, 0.42 g) and K_2CO_3 (32.0 mmol, 4.40 g) were added into a flask containing 120 mL 1,4-dioxane and 30 mL H_2O. After degassed four

times, the mixture solution was heated and refluxed under nitrogen for 4 hours. After cooling down to room temperature, the solvent was removed under reduced pressure and 100 mL water was added. The mixture was extracted using dichloromethane for three times. The organic phase was combined and dried over Na_2SO_4. The product was obtained after purifying by silica gel column chromatography as a yellow solid (5.43 g, yield: 85.6%). ^1H NMR (400 MHz, $CDCl_3$) δ ppm 8.03 (2H), 7.56 (2H), 7.15 (1H), 7.05 (1H), 4.38 (2H), 1.39 (3H).

Synthesis of diethyl 4,4'-(5,5'-(benzo[c][1,2,5]thiadiazole-4,7-diyl)bis(thiophene-5,2-diyl))dibenzoate

Ethyl 4-(5-bromothiophen-2-yl)benzoate (5.0 mmol, 1.55 g), 4,7-bis(4,4,5,5-tetramethyl-1,3,2-dioxaborolan-2-yl)benzo[c][1,2,5]thiadiazole (2.0 mmol, 0.78 g), $PdCl_2$ (0.2 mmol, 35.0 mg), PPh_3 (0.40 mmol, 0.10 g) and K_2CO_3 (8.0 mmol, 1.10 g) were added into 80 mL 1,4-dioxane and 20 mL H_2O. After degassed four times, the mixture solution was heated and refluxed under nitrogen for 16 hours. After cooling to room temperature, the solvent was removed by rotary evaporator. 100 mL water was added, and then the crude product was filtered and washed with water several times. The diethyl 4,4'-(5,5'-(benzo[c][1,2,5]thiadiazole-4,7-diyl)bis(thiophene-5,2-diyl))dibenzoate was obtained as a dark red solid (1.08 g, 93.8%). ^1H NMR (400 MHz, $CDCl_3$) δ ppm 8.13 (2H), 8.08 (4H), 7.94 (2H), 7.78 (4H), 7.53 (2H), 4.40 (4H), 1.41 (6H).

Synthesis of 4,4'-(5,5'-(benzo[c][1,2,5]thiadiazole-4,7-diyl)bis(thiophene-5,2-diyl))dibenzoic acid (BTTD)

Compound 6 (2.0 mmol, 1.15 g) was added into a mixture solution of 50 mL THF, 25 mL methanol and 25 mL with 2.5 g NaOH. The solution was heated to reflux at 80 °C overnight. After cooling to room temperature, the organic solvent was removed under reduced pressure.

Then the residue was neutralized using 2M HCl to obtain the precipitate, which was filtered to offer the final product BTTD as a dark red solid (0.96 g, 88.9%). ^{1}H NMR (400 MHz, DMSO-d_6) δ ppm 12.99 (2H), 8.23 (4H), 7.98 (4H), 7.88 (4H), 7.83 (2H).

Synthesis of ethyl 4-(4-bromothiophen-2-yl)benzoate

Compound ethyl 4-(4-bromothiophen-2-yl)benzoate was synthesized using the same route as ethyl 4-(5-bromothiophen-2-yl)benzoate. Ethyl 4-(4-bromothiophen-2-yl)benzoate was obtained as a white solid (5.23 g, yield: 84.6%). ^{1}H NMR (400 MHz, DMSO-d_6) δ ppm 7.95 (2H), 7.80 (2H), 7.77 (1H), 7.72 (1H), 4.29 (2H), 1.28 (3H).

Synthesis of diethyl 4,4'-(5,5'-(benzo[c][1,2,5]thiadiazole-4,7-diyl)bis(thiophene-5,3-diyl))dibenzoate

Compound diethyl 4,4'-(5,5'-(benzo[c][1,2,5]thiadiazole-4,7-diyl)bis(thiophene-5,3-diyl))dibenzoate was synthesized using the same route as diethyl 4,4'-(5,5'-(benzo[c][1,2,5]thiadiazole-4,7-diyl)bis(thiophene-5,2-diyl))dibenzoate. Diethyl 4,4'-(5,5'-(benzo[c][1,2,5]thiadiazole-4,7-diyl)bis(thiophene-5,3-diyl))dibenzoate was obtained as an orange solid (5.23 g, yield: 85.7%). ^{1}H NMR (400 MHz, $CDCl_3$) δ ppm 8.42 (2H), 8.11 (4H), 8.08 (2H), 7.93 (2H), 7.77 (4H), 4.40 (4H), 1.41 (6H).

Synthesis of 4,4'-(5,5'-(benzo[c][1,2,5]thiadiazole-4,7-diyl)bis(thiophene-5,3-diyl))dibenzoic acid (BTTB)

BTTB was synthesized using the same route as BTTD. BTTB was obtained as an orange (yield: 97.6%). 1H NMR (400 MHz, DMSO-d6) δ ppm 13.01 (2H), 8.70 (2H), 8.54 (2H), 8.27 (2H), 7.99 (4H), 7.90 (4H).

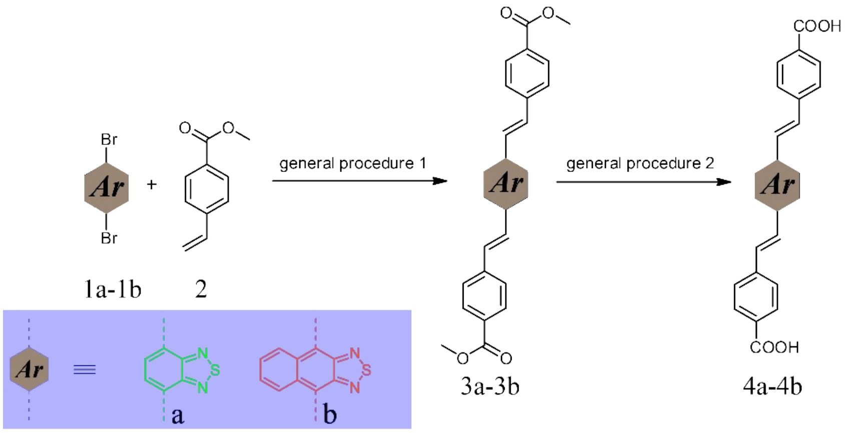

General procedure 1

Compound **1** (4.0 mmol), compound **2** (10.0 mmol, 1.62 g), $Pd(OAc)_2$ (0.1 mmol, 22.4 mg), NaOAc (20.0 mmol, 1.64 g) and n-Bu_4NBr (2.0 mmol, 0.65 g) were added in a flask containing 50 mL DMF. The reaction solution was degassed four times. Then the mixture was heated to 100°C for 24 h under nitrogen atmosphere. After cooling down to room temperature, 100 mL DI water was added. The crude product was obtained after filtration and washed using DI water, which was directly used for next step without further purification.

Dimethyl 4,4'-(1E,1'E)-2,2'-(benzo[*c*][1,2,5]thiadiazole-4,7-diyl)bis(ethene-2,1-diyl)dibenzoate (3a) was synthesized according to general procedure 1 (orange solid, yield: 94.8%). ^{1}H NMR (400 MHz, $CDCl_3$) δ 8.07 (6H), 7.71 (8H), 3.91 (6H).

Dimethyl 4,4'-(1E,1'E)-2,2'-(naphtho[2,3-*c*][1,2,5]thiadiazole-4,9-diyl)bis(ethene-2,1-diyl)dibenzoate (3b) was synthesized according to general procedure 1 (dark purple solid, yield: 97.8%). ^{1}H NMR (400 MHz, $CDCl_3$) δ 8.50 (2H), 8.31 (4H), 8.10 (4H), 7.79 (4H), 7.53 (2H), 3.95 (6H).

General procedure 2

4.0 mmol compound **3** was added to a solution containing 100 mL THF, 50 mL CH_3OH 50 mL water with 5.5 g NaOH. The mixture solution was heated to reflux at 80 °C overnight. After

cooling down to room temperature, the organic solvent was removed under reduced pressure. The resulted aqueous was neutralized using 2M HCl to obtain the precipitate, which was filtered to offer the final product **4**.

4,4'-(1E,1'E)-2,2'-(benzo[*c*][1,2,5]thiadiazole-4,7-diyl)bis(ethene-2,1-diyl)dibenzoic acid (4a, BTEBA) was synthesized according to general procedure 2 as an orange red solid (yield: 98.7%). ^{1}H NMR (400 MHz, DMSO-d_6) 8.14 (2H), 7.97 (6H), 7.81 (6H).

4,4'-(1E,1'E)-2,2'-(naphtho[2,3-*c*][1,2,5]thiadiazole-4,9-diyl)bis(ethene-2,1-diyl)dibenzoic acid (4b, NTEBA) was synthesized according to general procedure 2 as a dark purple solid (yield: 98.8%). ^{1}H NMR (400 MHz, DMSO-d_6) δ 8.64 (2H), 8.37 (4H), 7.94 (8H), 7.56 (2H). ^{13}C NMR (101 MHz, DMSO-d_6) 167.616, 150.857, 142.384, 137.044, 130.522, 130.268, 127.673, 126.001, 124.751, 124.598.

Synthesis of UiO-68-L (L: BIMB, BOMB, BTTA, BTAB, BTHB)

$ZrCl_4$ (11.2 mg, 0.048 mmol), organic linker (0.07 mmol) and benzoic acid (222.6 mg, 1.82 mmol) were added into a 5 mL vial containing 3 mL DMF. After sonicated for 5 minutes, the vial was put into a preheated oven at 120°C for 24 hours. After cooling down to room temperature, the octahedral single crystals were obtained, which was washed using DMF for several times until no fluorescent single was detected from the supernatant. The yields for UiO-68-BIMB, UiO-68-BOMB, UiO-68-BTTA, UiO-68-BTAB and UiO-68-BTHB are 76.3%, 67.6%, 89.2%, 69.7% and 72.5%.

Synthesis of HIAM-3001

$Zn(NO_3)_2\cdot 6H_2O$ (0.1 mmol, 29.7 mg), BDC (0.1 mmol, 16.6 mg) and ligand PBT (0.1 mmol, 29.0 mg) were added into a 5 mL vial containing 1 mL DMF and 3 mL H_2O. After sonicating

for 5 minutes, the capped the vial was put in a pre-heated oven to react at 100 ^{o}C for 3 days. The obtained single crystals were washed with DMF several times to remove unreacted ligand, and dried in the vacuum for characterization (yield: 71.8%).

Synthesis of HIAM-3002

$Zn(NO_3)_2·6H_2O$ (0.1 mmol, 29.7 mg), BDC (0.1 mmol, 16.6 mg) and ligand PNT (0.1 mmol, 34.0 mg) were added into a 5 mL vial containing 1 mL DMF and 3 mL H_2O. After sonicating for 5 minutes, the capped the vial was put in a pre-heated oven to react at 100 ^{o}C for 3 days. The obtained single crystals were washed with DMF several times to remove unreacted ligand, and dried in the vacuum for characterization (yield: 65.6%).

Synthesis of HIAM-3003

$Zn(NO_3)_2·6H_2O$ (0.1 mmol, 29.7 mg), BDC (0.1 mmol, 16.6 mg) and ligand PNS (0.1 mmol, 38.8 mg) were added into a 5 mL vial containing 1 mL DMF and 3 mL H_2O. After sonicating for 5 minutes, the capped the vial was put in a pre-heated oven to react at 100 ^{o}C for 3 days. The obtained single crystals were washed with DMF several times to remove unreacted ligand, and dried in the vacuum for characterization (yield: 68.4%).

Synthesis of HIAM-3004

$Zn(NO_3)_2·6H_2O$ (0.1 mmol, 29.7 mg) and ligand DPBT (0.1 mmol, 26.8 mg) were added into a 5 mL vial containing 1 mL DMF and 3 mL H_2O. After sonicating for 5 minutes, the capped the vial was put in a pre-heated oven to react at 100 ^{o}C for 3 days. The obtained single crystals were washed with DMF several times to remove unreacted ligand, and dried in the vacuum for characterization (yield: 83.8%).

Synthesis of HIAM-3005

$Zn(NO_3)_2 \cdot 6H_2O$ (0.1 mmol, 29.7 mg) and ligand DPBS (0.1 mmol,31.5 mg) were added into a 5 mL vial containing 1 mL DMF and 3 mL H_2O. After sonicating for 5 minutes, the capped the vial was put in a pre-heated oven to react at 100 ^oC for 3 days. The obtained single crystals were washed with DMF several times to remove unreacted ligand, and dried in the vacuum for characterization (yield: 80.9%).

Synthesis of HIAM-3006

$Zn(NO_3)_2 \cdot 6H_2O$ (0.1 mmol, 29.7 mg) and ligand DDPBT (0.1 mmol, 29.6 mg) were added into a 5 mL vial containing 1 mL DMF and 3 mL H_2O. After sonicating for 5 minutes, the capped the vial was put in a pre-heated oven to react at 100 ^oC for 3 days. The obtained single crystals were washed with DMF several times to remove unreacted ligand, and dried in the vacuum for characterization (yield: 76.6%).

Synthesis of HIAM-4005

L-proline (54.0 mg, 0.47 mmol) and concentrated HCl (63.0 μL) were added into a 5 mL vial, which was placed into a preheated oven at 100°C for 30 mins. After cooling down to room temperature, $ZrCl_4$ (22.0 mg, 0.094 mmol), BTEBA (40.0 mg, 0.094 mmol) and 3 mL DMF were added into the vial. After sonicated for 5 minutes, the vial was put into a preheated oven at 120°C for 48 hours. The vial was removed from the oven and allowed to cool to room temperature naturally. The orange octahedral single crystals were obtained and washed using DMF until no fluorescent single was detected from the supernatant (yield: 88.6%).

Synthesis of HIAM-4006

L-proline (54.0 mg, 0.47 mmol) and concentrated HCl (63.0 μL) were added into a 5 mL vial, which was placed into a preheated oven at 100°C for 30 mins. After cooling down to room

temperature, $ZrCl_4$ (22.0 mg, 0.094 mmol), NTEBA (45.0 mg, 0.094 mmol) and 3 mL DMF were added into the vial. After sonicated for 5 minutes, the vial was put into a preheated oven at 120°C for 48 hours. The vial was removed from the oven and allowed to cool to room temperature naturally. The dark red octahedral single crystals were obtained and washed using DMF until no fluorescent single was detected from the supernatant (yield: 82.6%).

Single-crystal X-ray diffraction analyses

Single crystals of HIAM-3002, HIAM-3003, HIAM-3004, HIAM-3006, HIAM-4005, HIAM-4006 were mounted on MicroMesh (MiTeGen) with paraton oil. All the data (except HIAM-3004) were collected on a 'Bruker D8 VENTURE' diffractometer equipped with copper micro-focus X-ray sources (λ = 1.5406 Å), and that of HIAM-3004 was measured on a 'Bruker D8 VENTURE' diffractometer equipped with gallium micro-focus metaljet X-ray sources (λ = 1.34050 Å). The crystals kept at the 298K (HIAM-3002), 200K (HIAM-3003), 190K (HIAM-3004), 293K (HIAM-3006), 200K (HIAM-4005) and 200K (HIAM-4006) during data collection. Using Olex2[6], the structures were solved with the ShelXT[7] structure solution program using Intrinsic Phasing and refined with the ShelXL[8] refinement package using Least Squares minimization. Because ligands were disordered in the structures (the naphtho[2,3-c][1,2,5]selenadiazole group of the PNS in HIAM-3003, the DDPBT in HIAM-3006, the benzo[c][1,2,5]thiadiazole group of the BTEBA in HIAM-4005, and the naphtho[2,3-c][1,2,5]thiadiazole group of the NTEBA in HIAM-4006) , atoms were refined using geometry restrains (SADI, DFIX, FLAT), and restraints were also used to refine anisotropic displacement parameters of all non-hydrogen atoms (SIMU and DELU). The hydrogen atoms on the aromatic rings were located at geometrically calculated positions and refined by riding. However, the

hydrogen atoms for the coordinated molecules cannot be found from the residual electron density peaks and the attempt of theoretical addition was not done. The free solvent molecules are highly disordered in MOFs, and attempts to locate and refine the solvent peaks were unsuccessful. The diffused electron densities resulting from these solvent molecules were removed using the SQUEEZE routine of PLATON[9]; structures were then refined again using the data generated. The refinement results are summarized in Table S1-S6. Crystallographic data for all of the crystal structures in CIF format have been deposited in the Cambridge Crystallographic Data Centre (CCDC) under deposition numbers CCDC-2129032 (HIAM-3002), CCDC-2129033 (HIAM-3003), CCDC-2129034 (HIAM-3004), CCDC-2129035 (HIAM-3006), CCDC-2129036 (HIAM-4005), CCDC-2129037 (HIAM-4006). The data can be obtained free of charge via www.ccdc.cam.ac.uk/data_request/cif (or from the Cambridge Crystallographic Data Centre, 12 Union Road, Cambridge CB2 1EZ, U.K.)

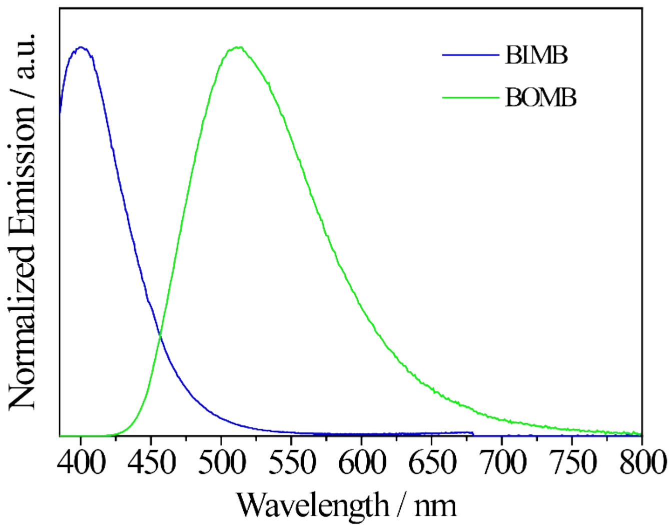

Figure S1. The normalized emission spectra of BIMB and BOMB under 365 nm excitation.

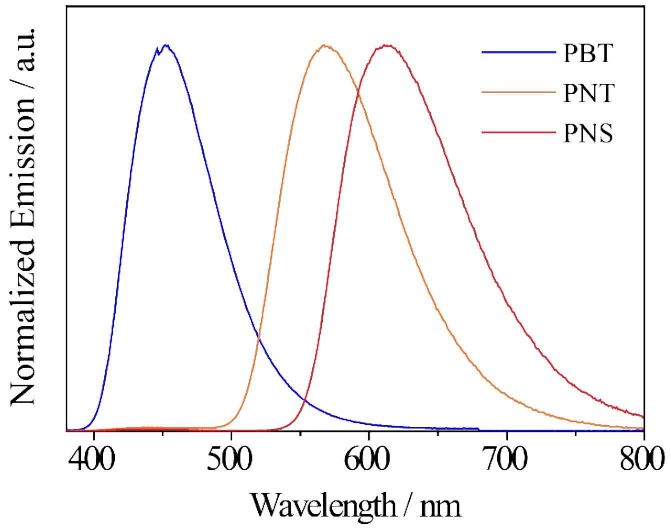

Figure S2. The normalized emission spectra of PBT, PNT and PNS under 365 nm excitation.

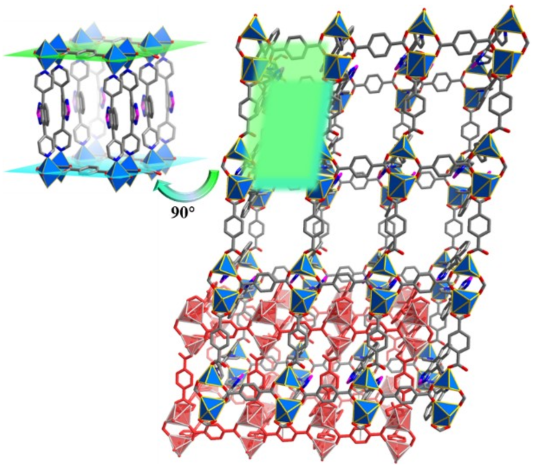

Figure S3. The crystal structure of HIAM-3003.

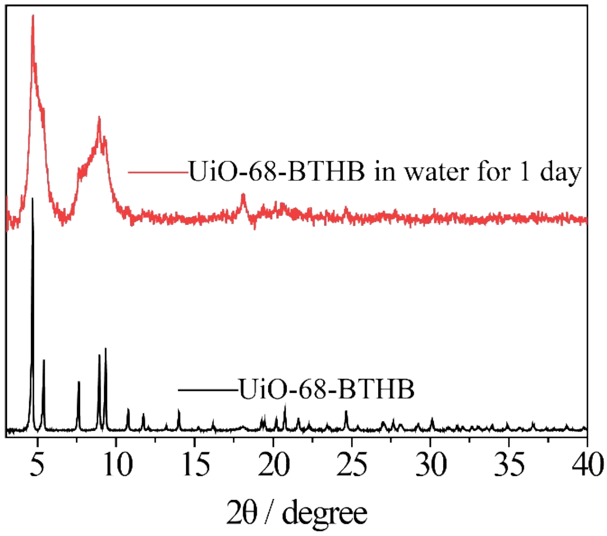

Figure S4. The PXRD patterns of the as-synthesized UiO-68-BTHB and sample after treatment in water for one day.

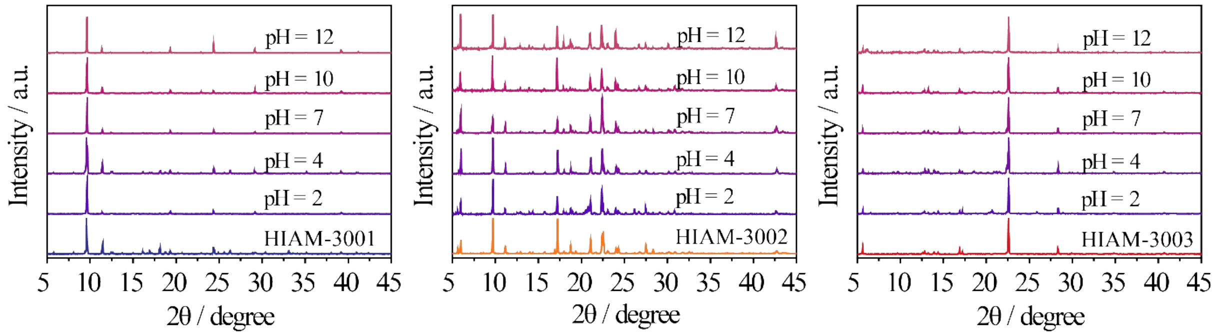

Figure S5. The PXRD patterns of the as-synthesized HIAM-3001, HIAM-3002 and HIAM-3003, and samples after treatment under different conditions for one day.

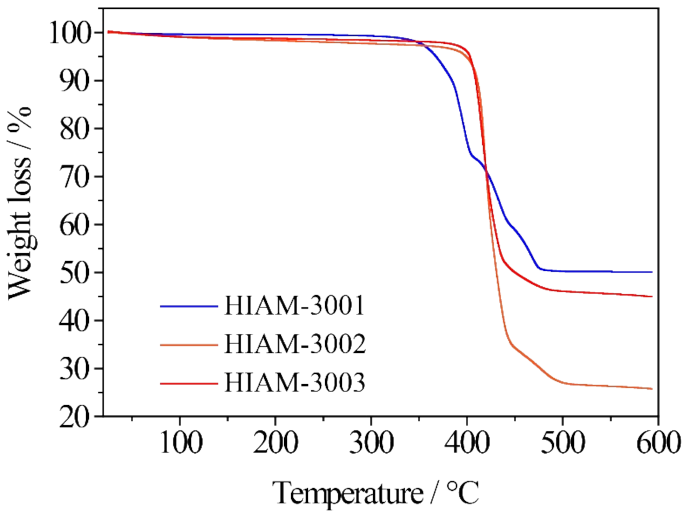

Figure S6. The TGA profiles of HIAM-3001, HIAM-3002 and HIAM-3003.

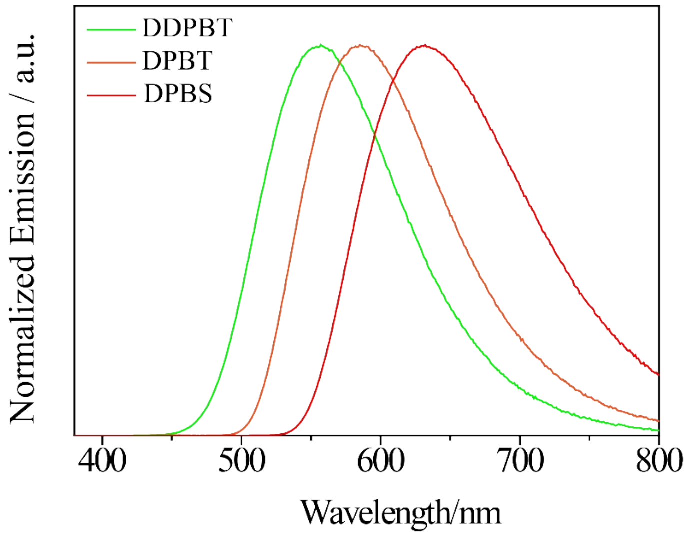

Figure S7. The normalized emission spectra of DDPBT, DPBT and DPBS under 365 nm excitation.

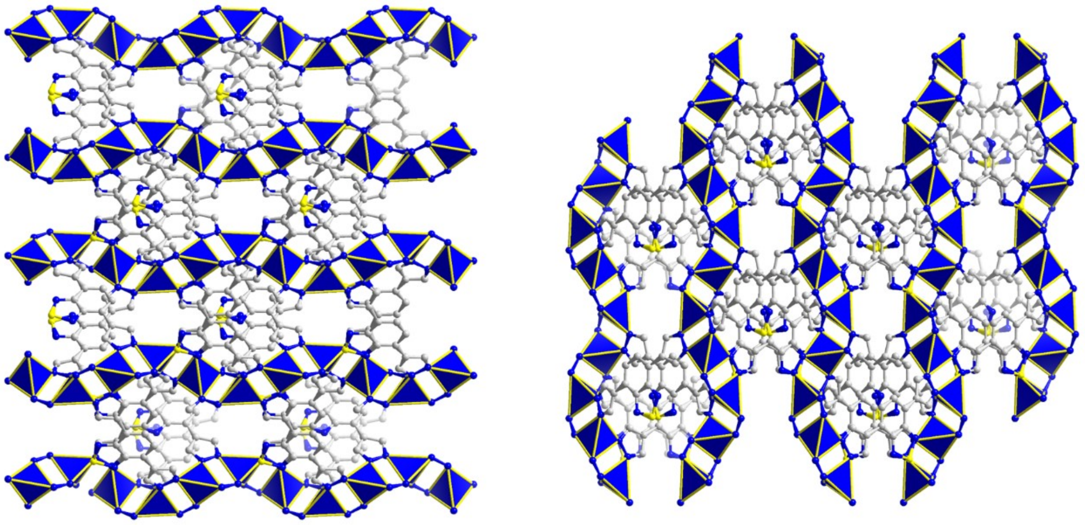

Figure S8. The crystal structure of HIAM-3004 viewed along *a* (left) and *b* (right) axis.

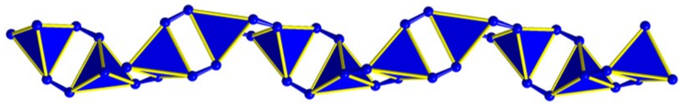

Figure S9. The 1D helical chain in HIAM-3004 along the *c* axis.

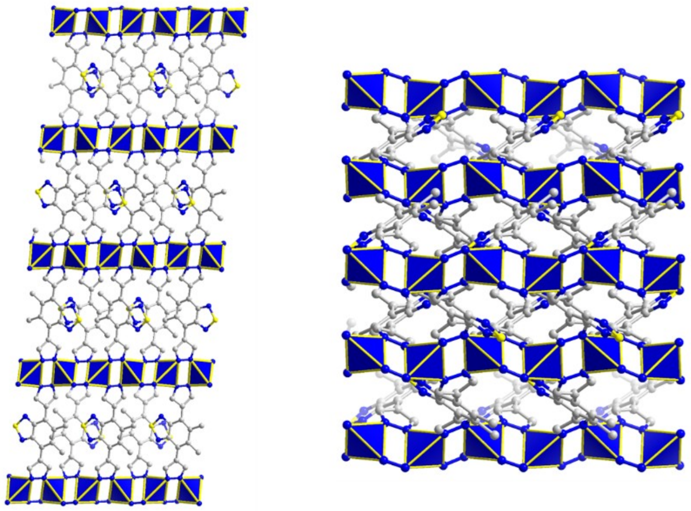

Figure S10. The crystal structure of HIAM-3006 viewed along *b* (left) and *c* (right) axis.

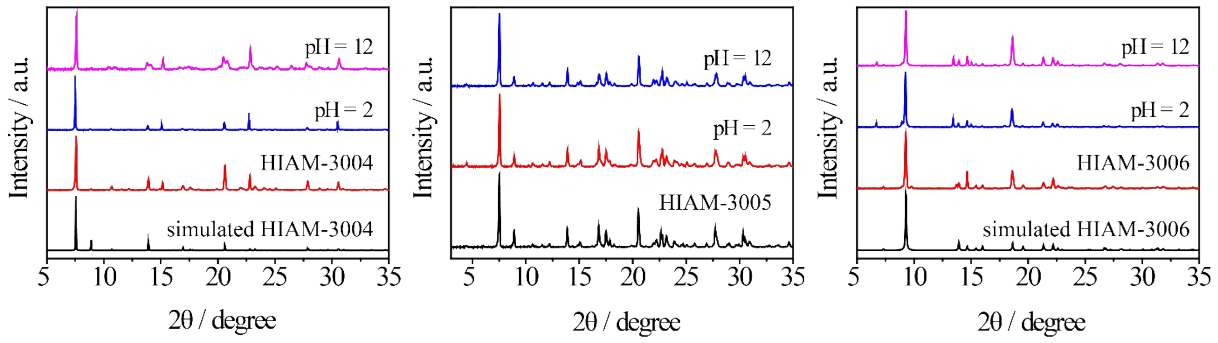

Figure S11. The PXRD patterns of the as-synthesized HIAM-3004, HIAM-3005 and HIAM-3006, and samples after treatment under different conditions for one day.

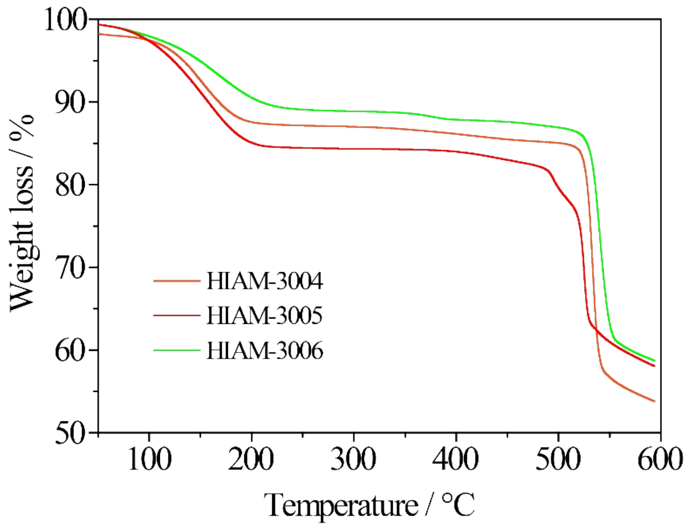

Figure S12. The TGA curves of HIAM-3004, HIAM-3005 and HIAM-3006.

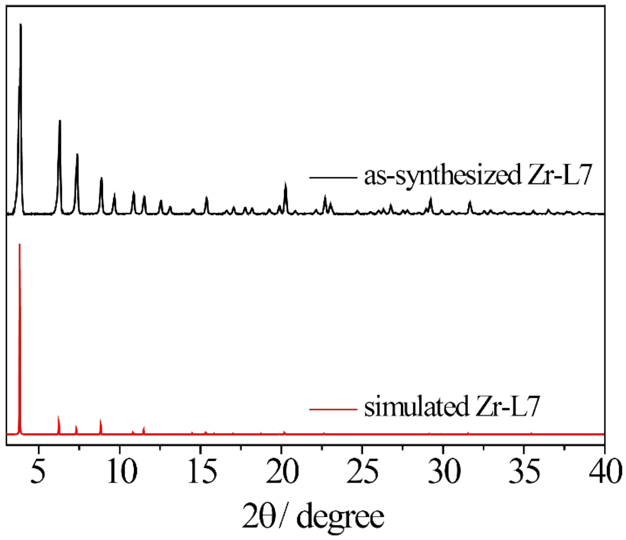

Figure S13. The PXRD patterns of the simulated and as-synthesized Zr-L7.

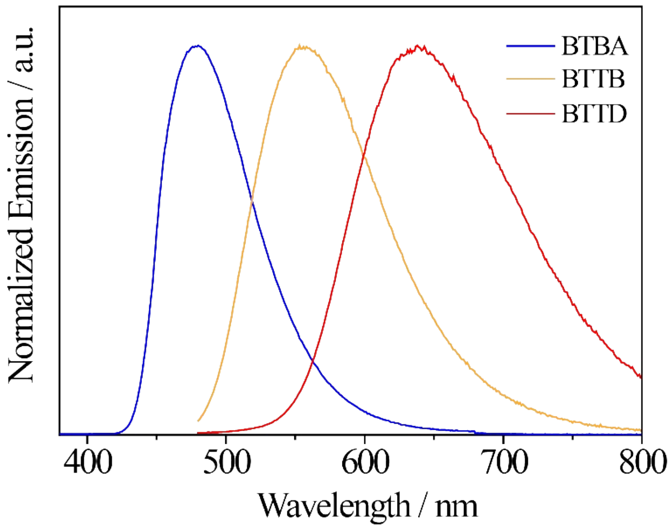

Figure S14. The normalized emission spectra of BTBA, BTTB and BTTD under 365 nm excitation.

Table S1. Crystal data and structure refinement details for HIAM-3002.

CCDC No.	2129032	
Empirical formula	C112H64N16O17S4Zn4	
Formula weight	2295.51	
Temperature	298 K	
Wavelength	1.54178 Å	
Crystal system	triclinic	
Space group	P-1	
Unit cell dimensions	a = 10.4363(8) Å	α = 88.157(3)°
	b = 15.0079(11) Å	β = 88.133(3)°
	c = 15.7667(10) Å	γ = 81.780(3)°
Volume	2441.9(3) $Å^3$	
Z	1	
Density (calculated)	1.561 g/cm^3	
Absorption coefficient	2.584 mm^{-1}	
F(000)	1168.0	
Crystal size	0.15 x 0.15 x 0.08 mm^3	
Theta range for data collection	5.952 to 133.196°	
Index ranges	-12<=h<=12, -17<=k<=17, 0<=l<=18	
Reflections collected	8489	
Independent reflections	8489 [R(int) = 0.0704, R(sigma) = 0.0466]	
Data / restraints / parameters	8489 / 0 / 696	
Goodness-of-fit on F^2	1.040	
Final R indices [I>2sigma(I)]	R1 = 0.0451, wR2 = 0.1282	
R indices (all data)	R1 = 0.0493, wR2 = 0.1326	
Largest diff. peak and hole	0.65 and -0.52 e.$Å^{-3}$	

Table S2. Crystal data and structure refinement details for HIAM-3003.

CCDC No.	2129033	
Empirical formula	C112H64N16O17Se4Zn4	
Formula weight	2483.11	
Temperature	200 K	
Wavelength	1.54178 Å	
Crystal system	triclinic	
Space group	P-1	
Unit cell dimensions	a = 10.4136(3) Å	α = 88.538(2)°
	b = 15.0054(4) Å	β = 87.9310(10)°
	c = 15.7481(4) Å	γ = 81.4230(10)°
Volume	2431.19(11) $Å^3$	
Z	1	
Density (calculated)	1.696 g/cm^3	
Absorption coefficient	3.505 mm^{-1}	
F(000)	1240.0	
Crystal size	0.6 x 0.2 x 0.02 mm^3	
Theta range for data collection	5.616 to 133.432°	
Index ranges	-10<=h<=12, -17<=k<=17, -18<=l<=18	
Reflections collected	30102	
Independent reflections	8587 [R(int) = 0.0626, R(sigma) = 0.0558]	
Data / restraints / parameters	8587 / 1185 / 691	
Goodness-of-fit on F^2	1.079	
Final R indices [I>2sigma(I)]	R1 = 0.0732, wR2 = 0.1913	
R indices (all data)	R1 = 0.0871, wR2 = 0.1998	
Largest diff. peak and hole	0.97 and -0.98 e.$Å^{-3}$	

Table S3. Crystal data and structure refinement details for HIAM-3004.

CCDC No.	2129034	
Empirical formula	C12H6N6SZn	
Formula weight	331.66	
Temperature	190 K	
Wavelength	1.34139 Å	
Crystal system	tetragonal	
Space group	$I4_1$	
Unit cell dimensions	a = 16.5490(14) Å	α = 90°
	b = 16.5490(14) Å	β = 90°
	c = 12.4563 (12) Å	γ = 90°
Volume	3411.4 (7) $Å^3$	
Z	8	
Density (calculated)	1.292 g/cm^3	
Absorption coefficient	2.071 mm^{-1}	
F(000)	1328.0	
Crystal size	0.12 x 0.1 x 0.1 mm^3	
Theta range for data collection	6.572 to 107.732°	
Index ranges	-14<=h<=14, 0<=k<=19, -14<=l<=14	
Reflections collected	2887	
Independent reflections	2887 [R(int) = 0.0798, R(sigma) = 0.0777]	
Data / restraints / parameters	0887 / 174 / 170	
Goodness-of-fit on F^2	1.093	
Final R indices [I>2sigma(I)]	R1 = 0.0718, wR2 = 0.1839	
R indices (all data)	R1 = 0.0770, wR2 = 0.1932	
Largest diff. peak and hole	1.62 and -0.61 e.$Å^{-3}$	

Table S4. Crystal data and structure refinement details for HIAM-3006.

CCDC No.	2129035	
Empirical formula	C14H10N6SZn	
Formula weight	359.71	
Temperature	293 K	
Wavelength	1.54178 Å	
Crystal system	monoclinic	
Space group	$P2_1/n$	
Unit cell dimensions	a = 7.0203(2) Å	α = 90°
	b = 10.3506(3) Å	β = 95.4420(10)°
	c = 24.2607(7) Å	γ = 90°
Volume	1754.94(9) $Å^3$	
Z	4	
Density (calculated)	1.361 g/cm^3	
Absorption coefficient	3.091 mm^{-1}	
F(000)	728.0	
Crystal size	0.15 x 0.08 x 0.06 mm^3	
Theta range for data collection	11.258 to 144.218°	
Index ranges	-8<=h<=8, -12<=k<=12, -29<=l<=28	
Reflections collected	17460	
Independent reflections	3455 [R(int) = 0.0486, R(sigma) = 0.0363]	
Data / restraints / parameters	3455/ 945 / 339	
Goodness-of-fit on F^2	1.064	
Final R indices [I>2sigma(I)]	R1 = 0.0393, wR2 = 0.1123	
R indices (all data)	R1 = 0.0470, wR2 = 0.1187	
Largest diff. peak and hole	0.26 and -0.42 e.$Å^{-3}$	

Table S5. Crystal data and structure refinement details for HIAM-4005.

CCDC No.	2129036	
Empirical formula	C144H82N12O32S6Zr6	
Formula weight	3231.89	
Temperature	200 K	
Wavelength	1.54178 Å	
Crystal system	cubic	
Space group	Fd-3m	
Unit cell dimensions	a = 39.3006(5) Å	α = 90°
	b = 39.3006(5) Å	β = 90°
	c = 39.3006(5) Å	γ = 90°
Volume	60701(2) $Å^3$	
Z	8	
Density (calculated)	0.707 g/cm^3	
Absorption coefficient	2.325 mm^{-1}	
F(000)	12976.0	
Crystal size	0.08 x 0. 08 x 0. 08 mm^3	
Theta range for data collection	6.36 to 136.618°	
Index ranges	-38<=h<=32, -44<=k<=42, -46<=l<=36	
Reflections collected	13875	
Independent reflections	2616 [R(int) = 0.0364, R(sigma) = 0.0257]	
Data / restraints / parameters	2616 / 150 / 167	
Goodness-of-fit on F^2	1.122	
Final R indices [I>2sigma(I)]	R1 = 0.0528, wR2 = 0.1621	
R indices (all data)	R1 = 0.0608, wR2 = 0.1732	
Largest diff. peak and hole	1.04 and -0.76 e.$Å^{-3}$	

Table S6. Crystal data and structure refinement details for HIAM-4006.

CCDC No.	2129037	
Empirical formula	C7H3.83N0.5O1.33S0.25Zr0.25	
Formula weight	147.09	
Temperature	299 K	
Wavelength	1.54178 Å	
Crystal system	cubic	
Space group	Fd-3m	
Unit cell dimensions	a = 39.2392(5) Å	α = 90°
	b = 39.2392(5) Å	β = 90°
	c = 39.2392(5) Å	γ = 90°
Volume	60417(2) $Å^3$	
Z	8	
Density (calculated)	0.776 g/cm^3	
Absorption coefficient	2.365 mm^{-1}	
F(000)	14208.0	
Crystal size	0.05 x 0. 05 x 0. 05 mm^3	
Theta range for data collection	6.37 to 136.49°	
Index ranges	-42<=h<=47, -35<=k<=45, -37<=l<=47	
Reflections collected	36246	
Independent reflections	2635 [R(int) = 0.0548, R(sigma) = 0.0195]	
Data / restraints / parameters	2635 / 96/ 132	
Goodness-of-fit on F^2	1.097	
Final R indices [I>2sigma(I)]	R1 = 0.0409, wR2 = 0.1194	
R indices (all data)	R1 = 0.0478, wR2 = 0.1286	
Largest diff. peak and hole	0.48 and -0.76 e.$Å^{-3}$	

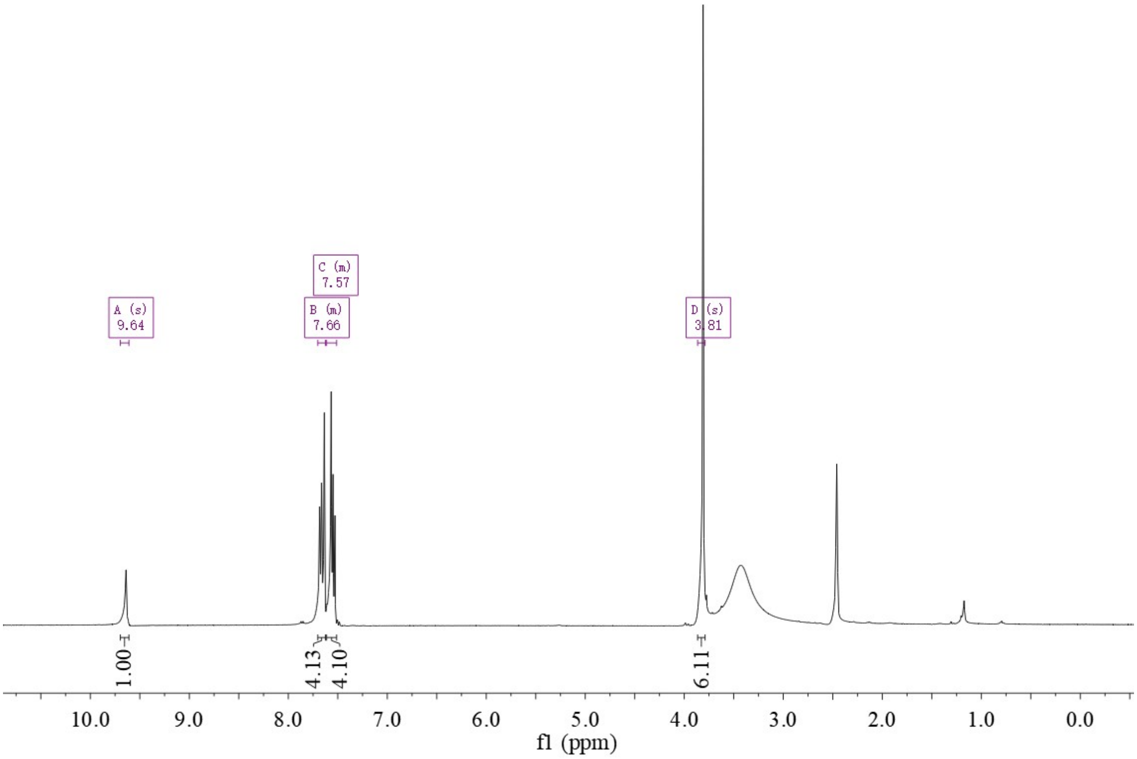

Figure S15. ^{1}H NMR spectrum of BIMB in DMSO-d_6.

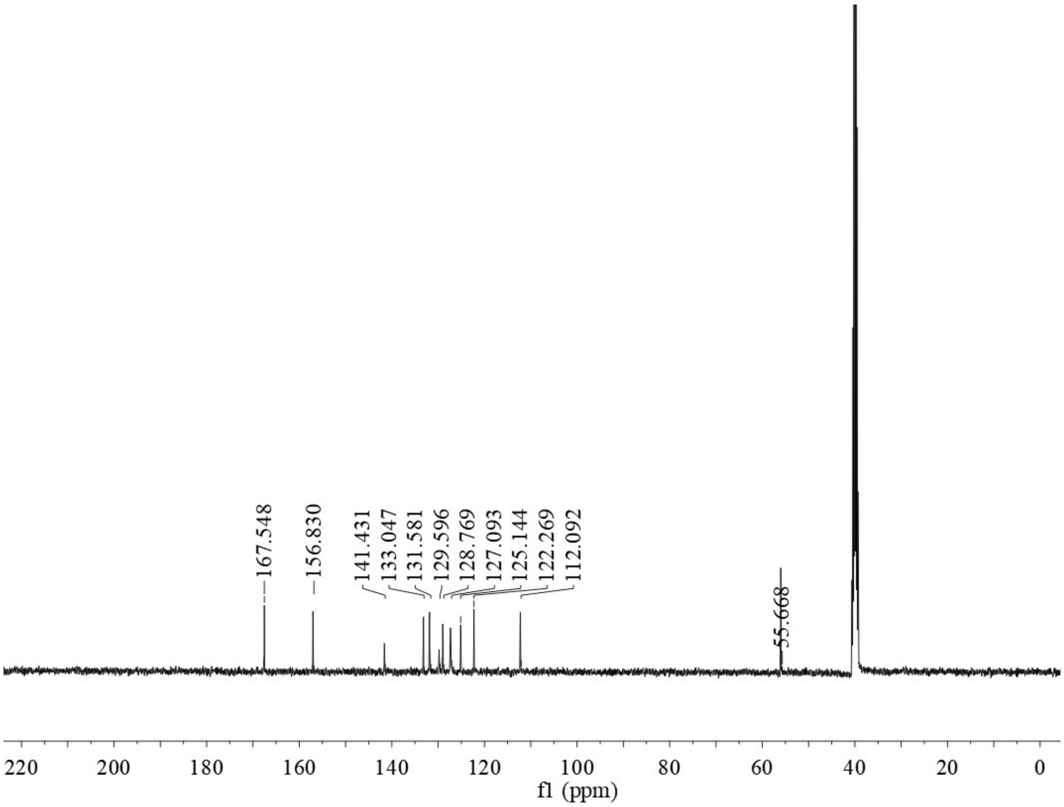

Figure S16. ^{13}C NMR spectrum of BIMB in DMSO-d_6.

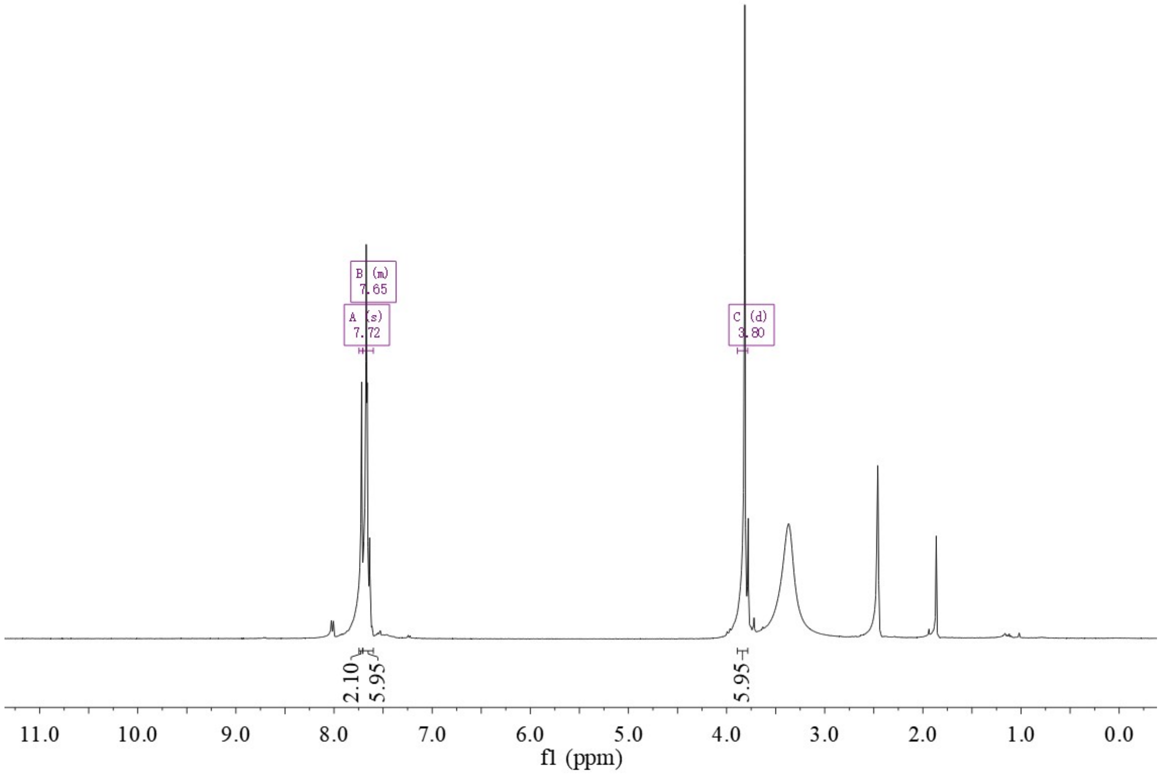

Figure S17. ^{1}H NMR spectrum of BOMB in DMSO-d_6.

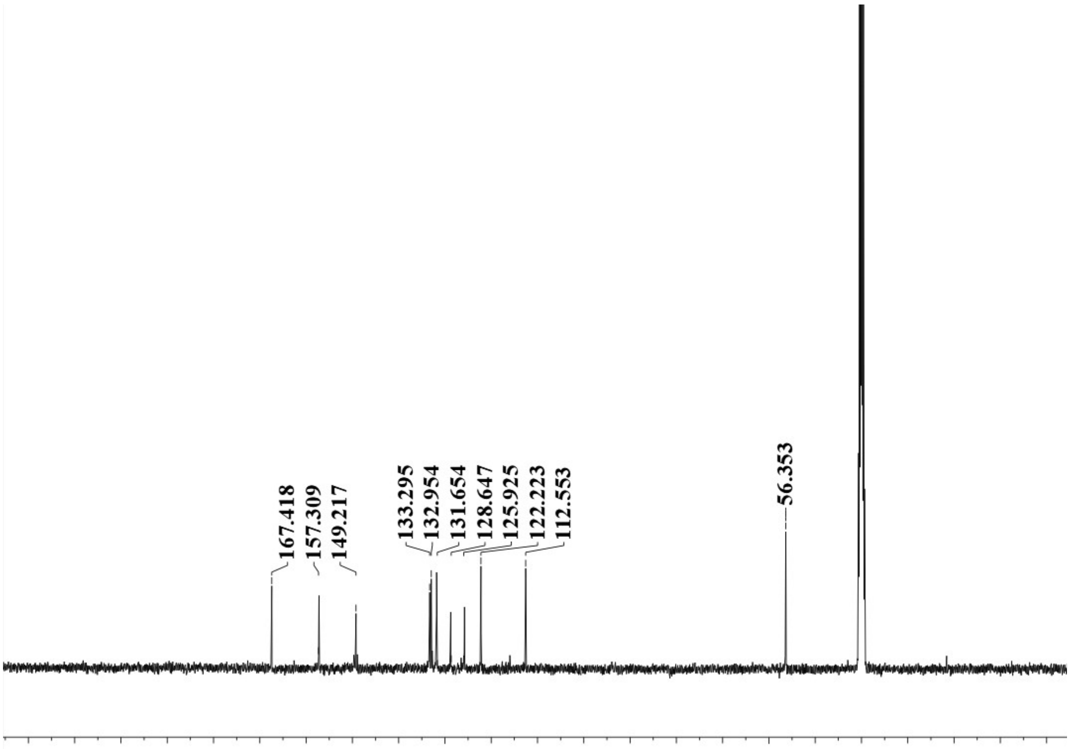

Figure S18. ^{13}C NMR spectrum of BOMB in DMSO-d_6.

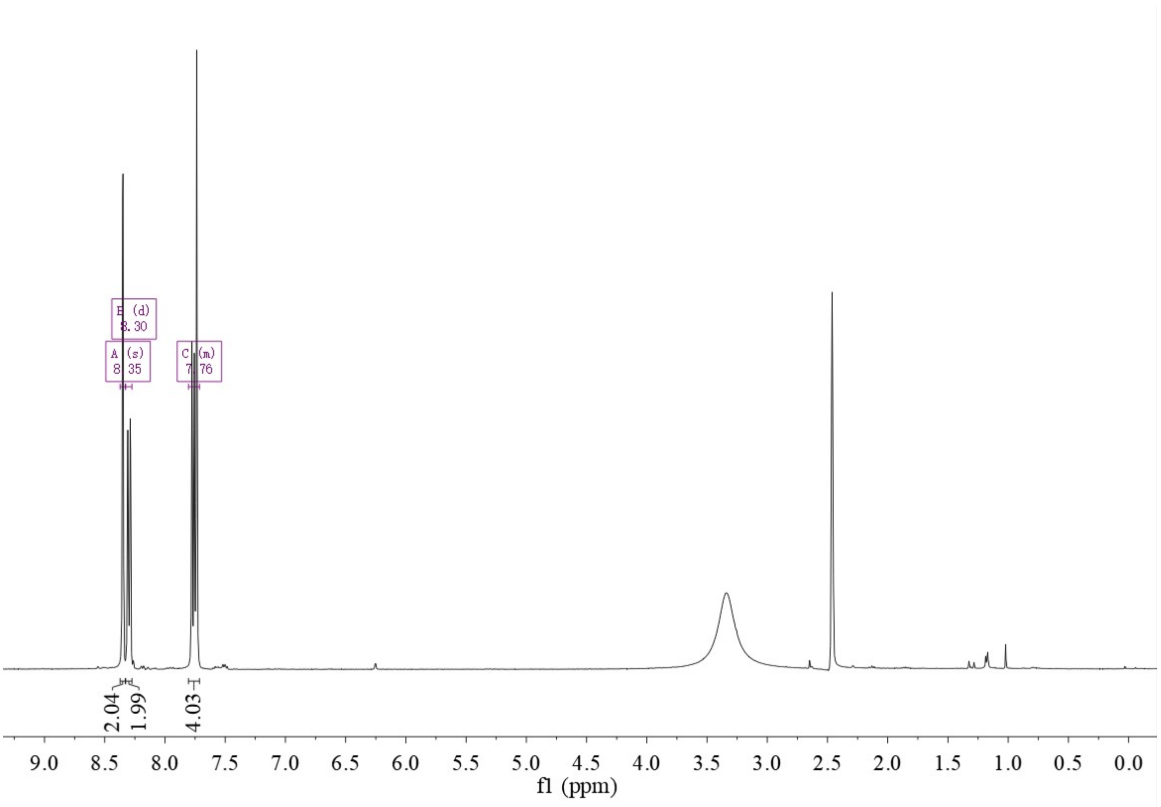

Figure S19. ^{1}H NMR spectrum of BTTB in DMSO-d_6.

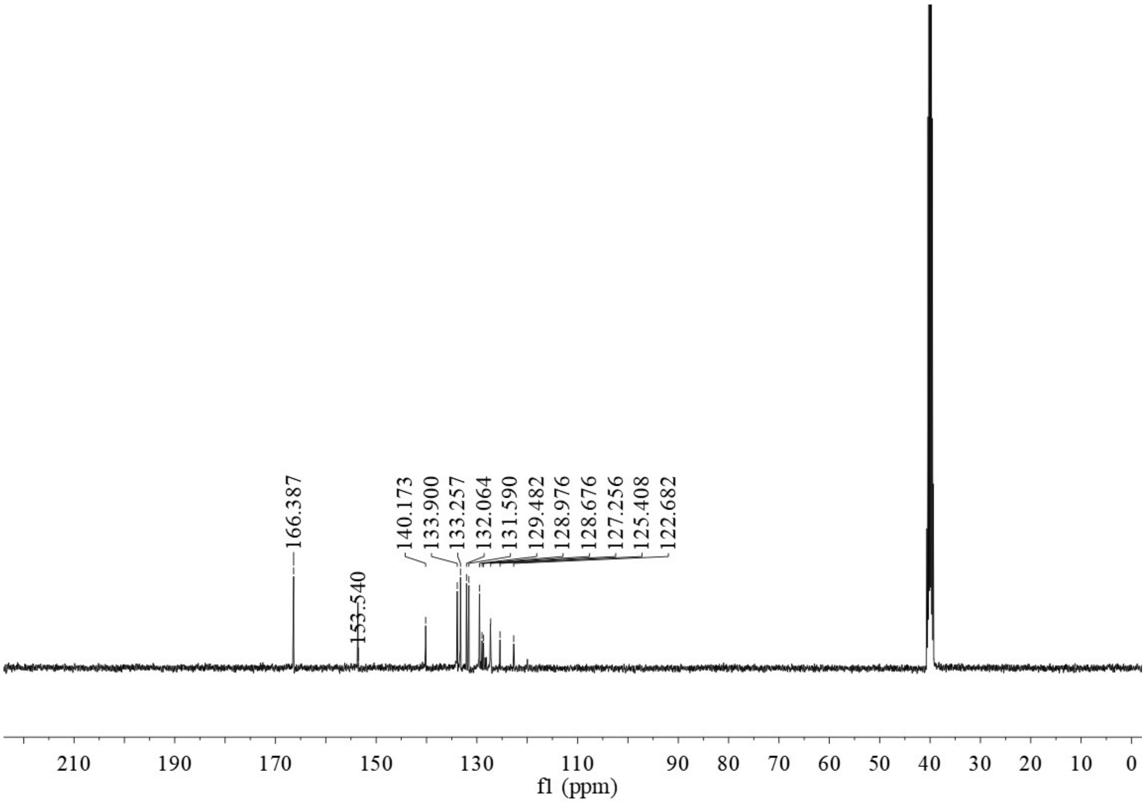

Figure S20. ^{13}C NMR spectrum of BTTB in DMSO-d_6.

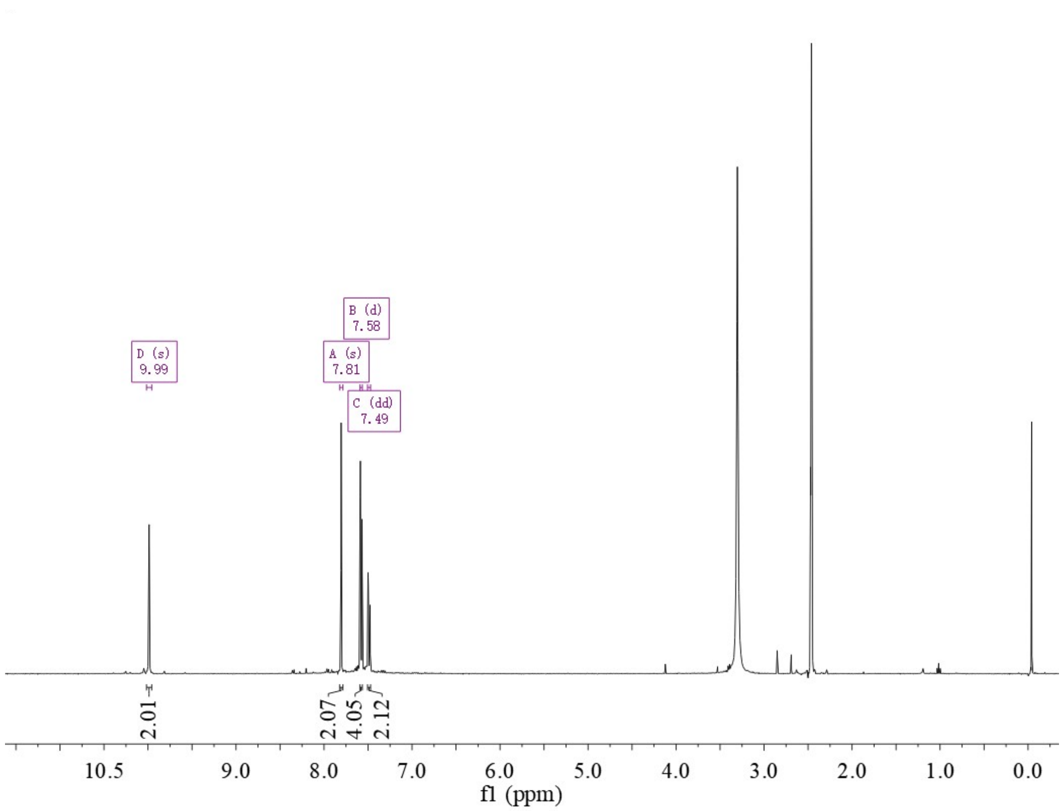

Figure S21. ^{1}H NMR spectrum of BTHB in DMSO-d_6.

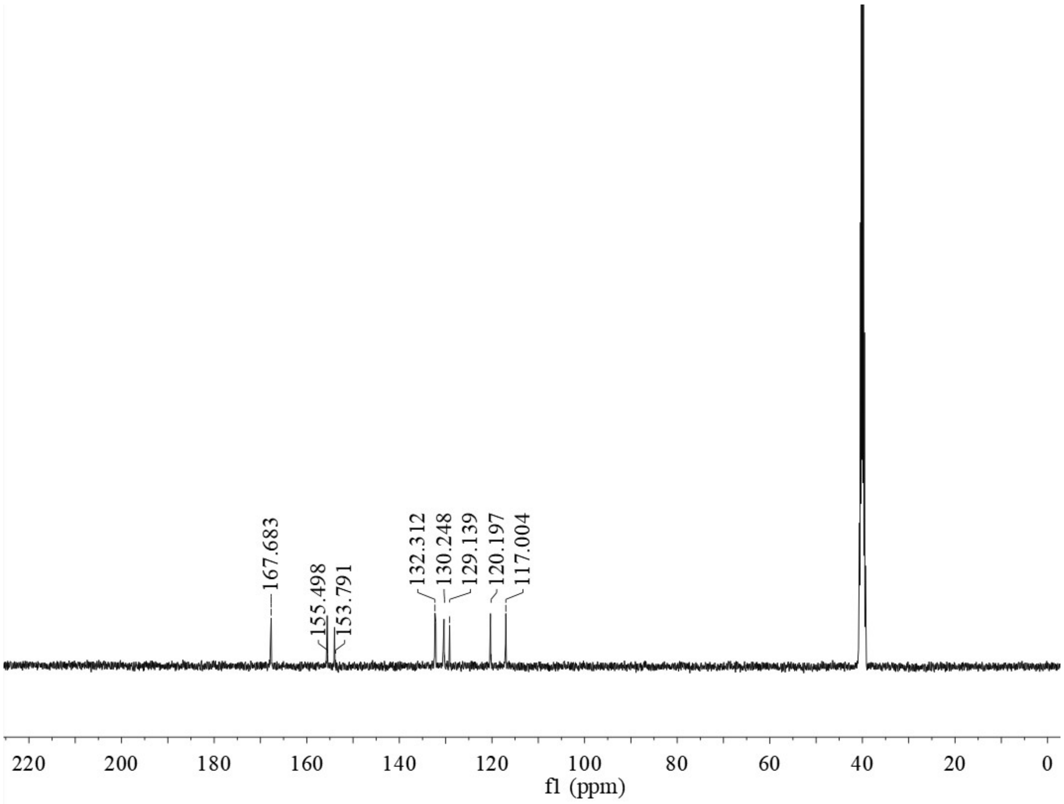

Figure S22. ^{13}C NMR spectrum of BTHB in DMSO-d_6.

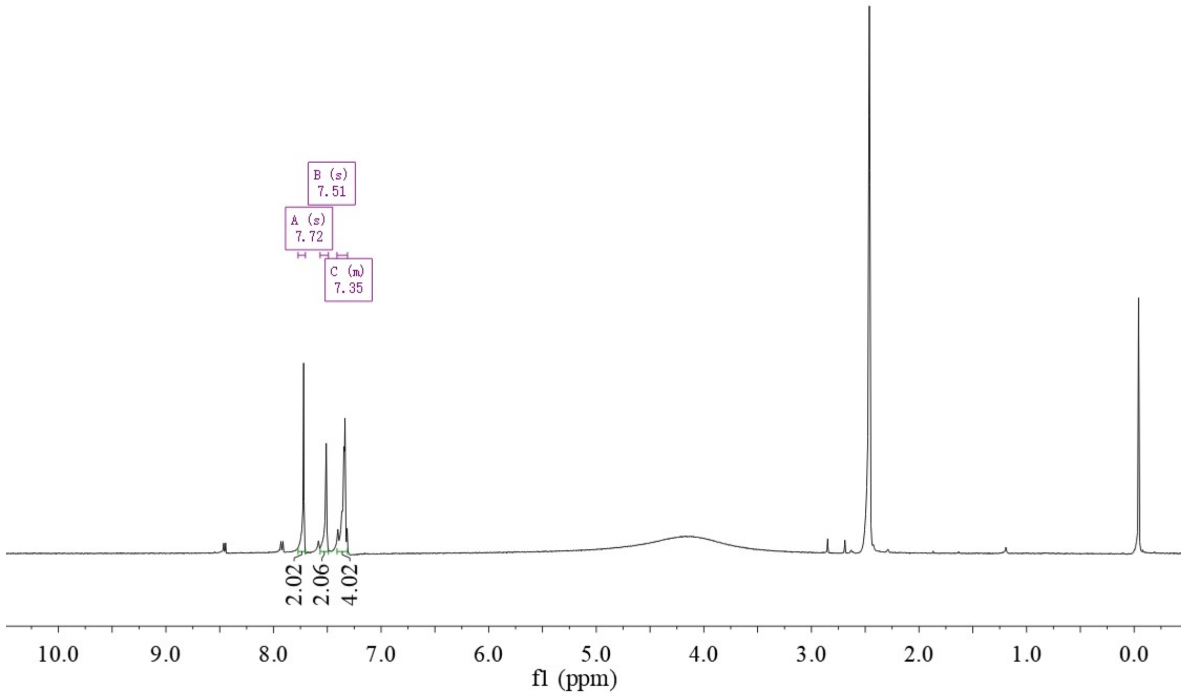

Figure S23. ^{1}H NMR spectrum of BTAB in DMSO-d_6.

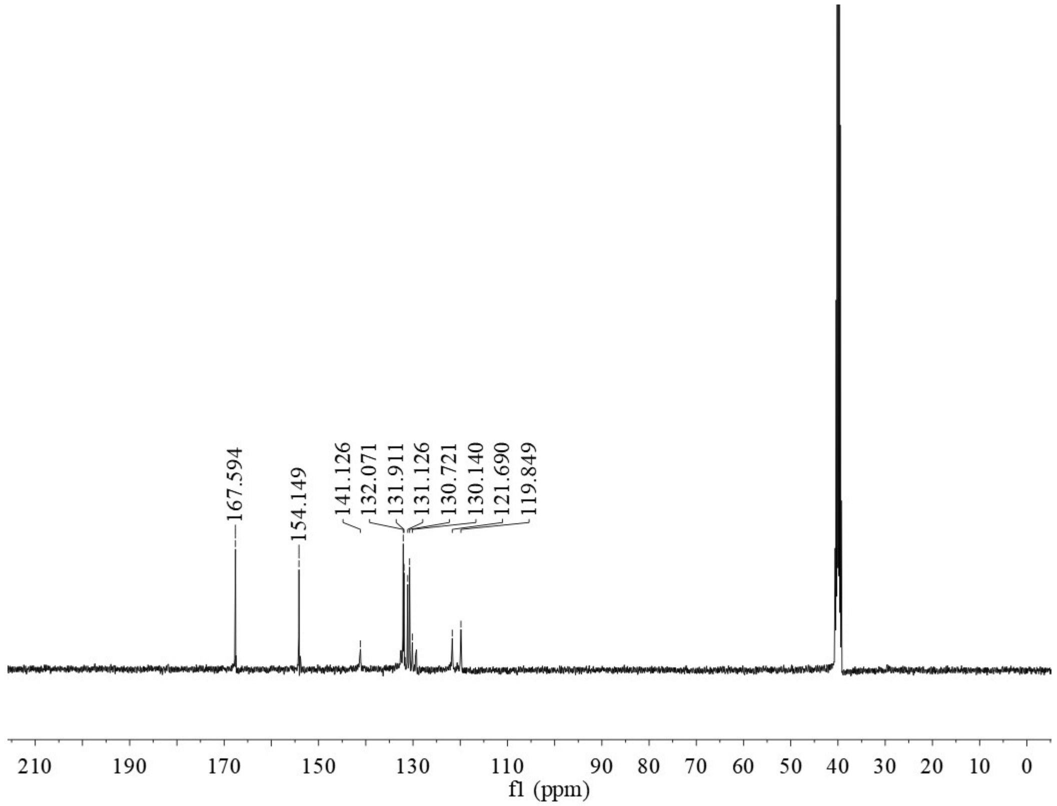

Figure S24. ^{13}C NMR spectrum of BTAB in DMSO-d_6.

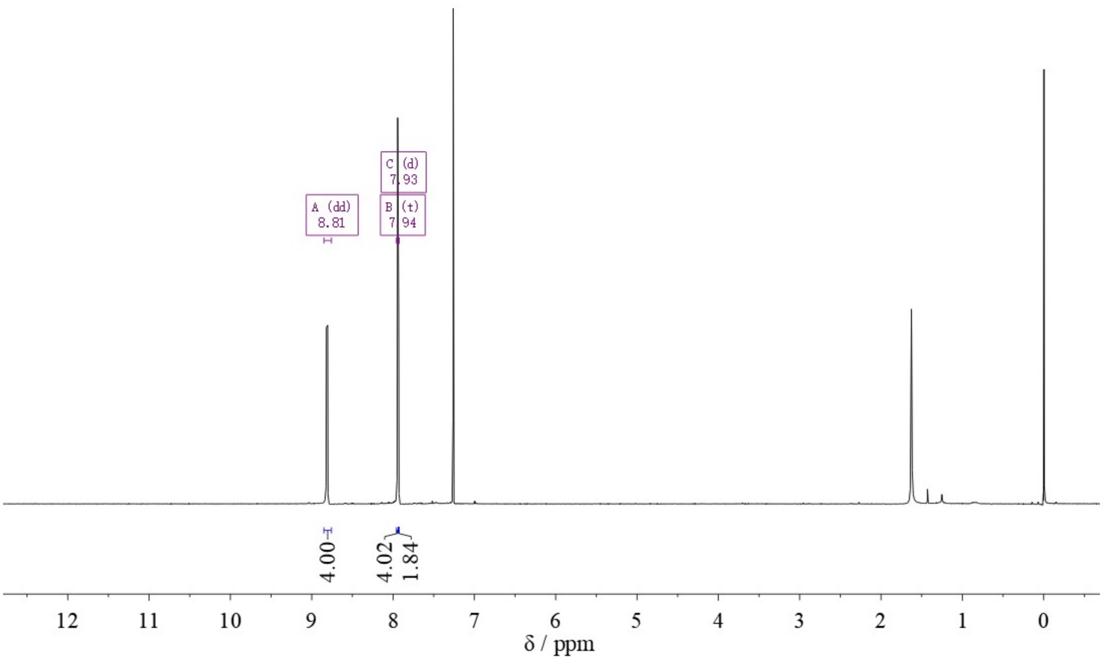

Figure S25. ^{1}H NMR spectrum of PBT in $CDCl_3$.

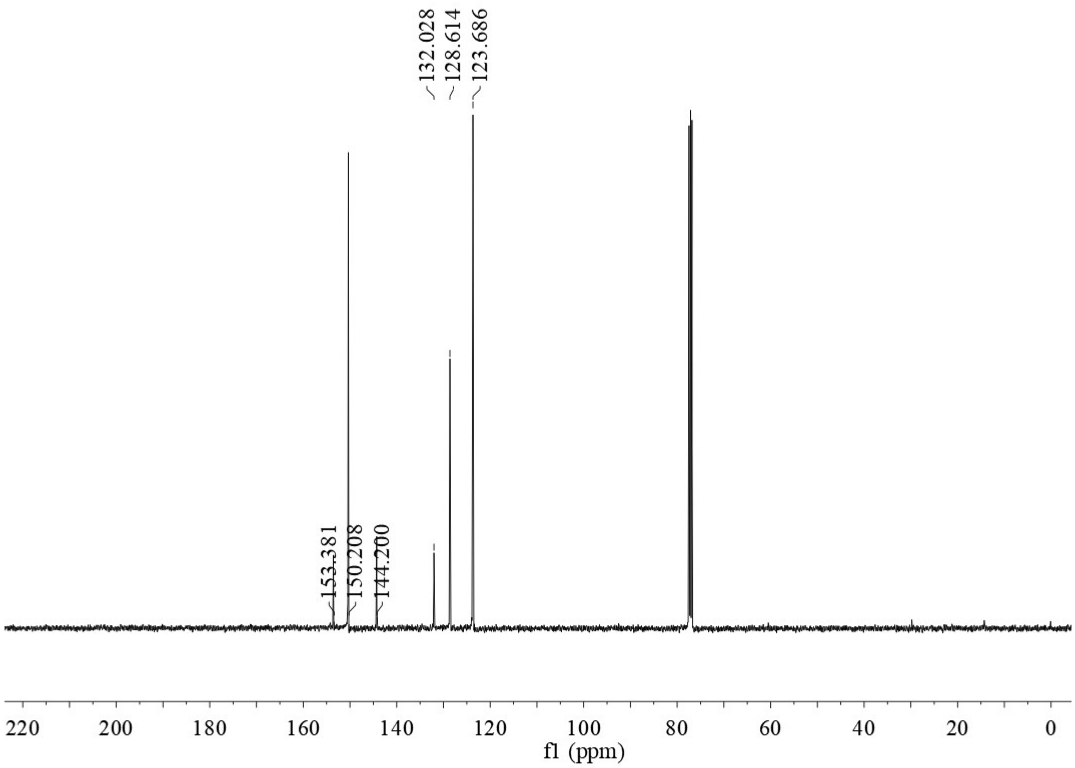

Figure S26. ^{13}C NMR spectrum of PBT in $CDCl_3$.

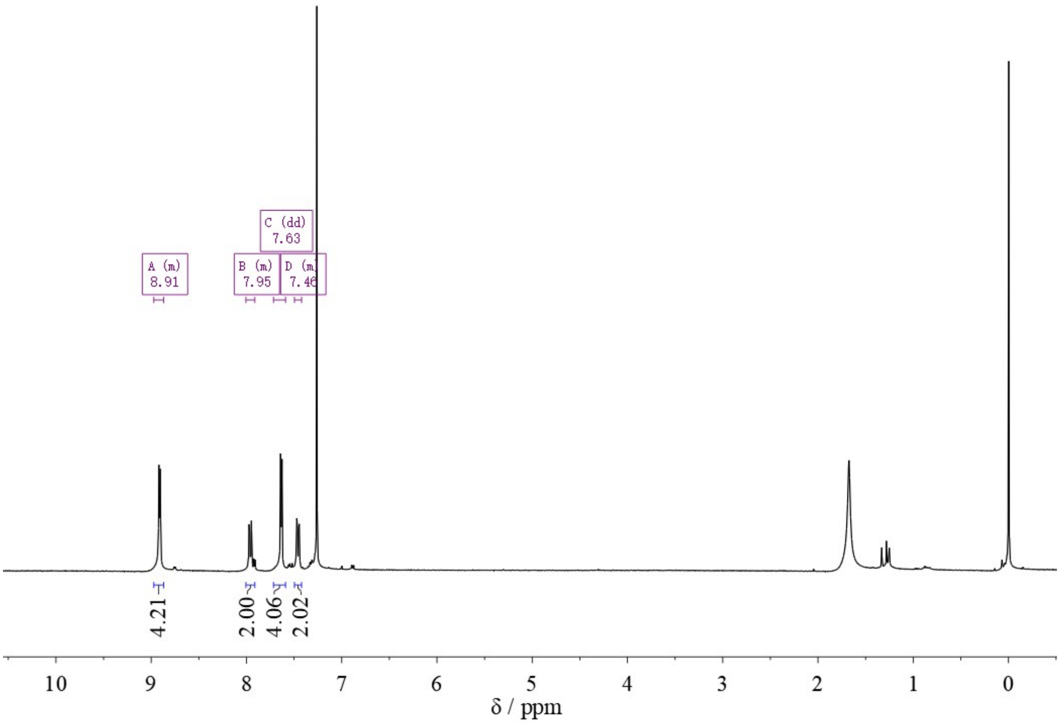

Figure S27. ^{1}H NMR spectrum of PNT in $CDCl_3$.

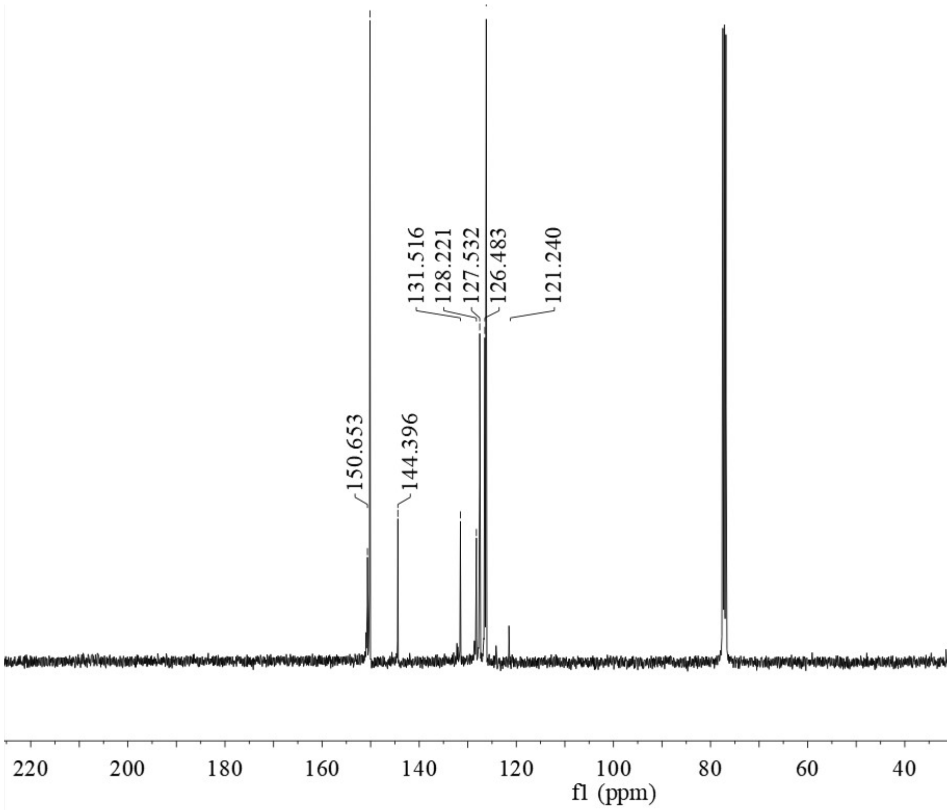

Figure S28. ^{13}C NMR spectrum of PNT in $CDCl_3$.

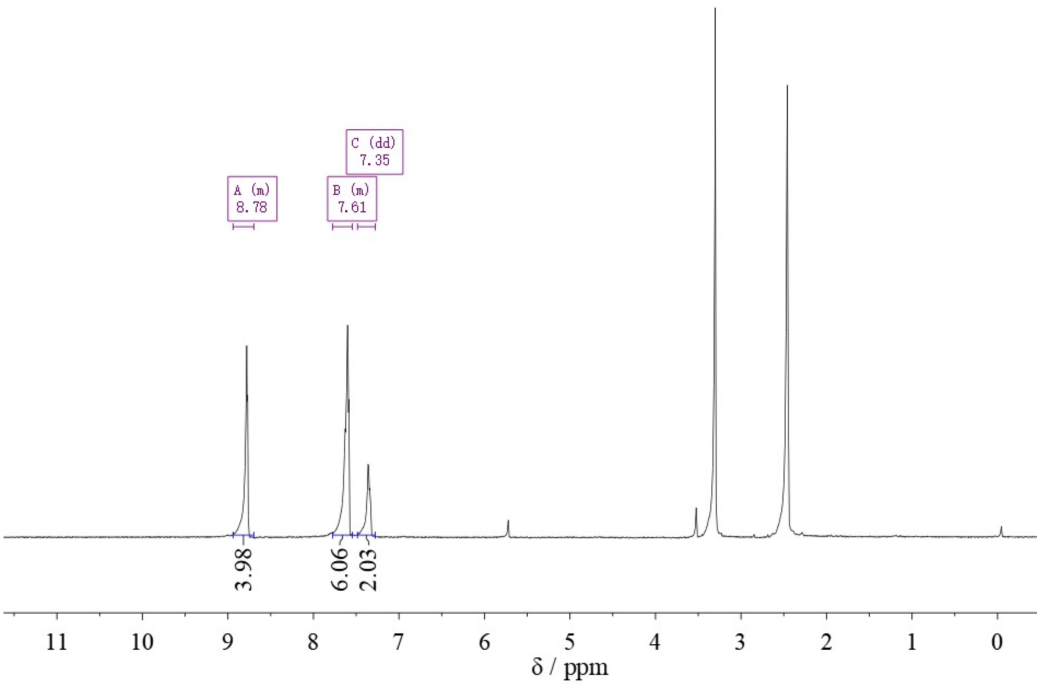

Figure S29. ^{1}H NMR spectrum of PNS in DMSO-d_6.

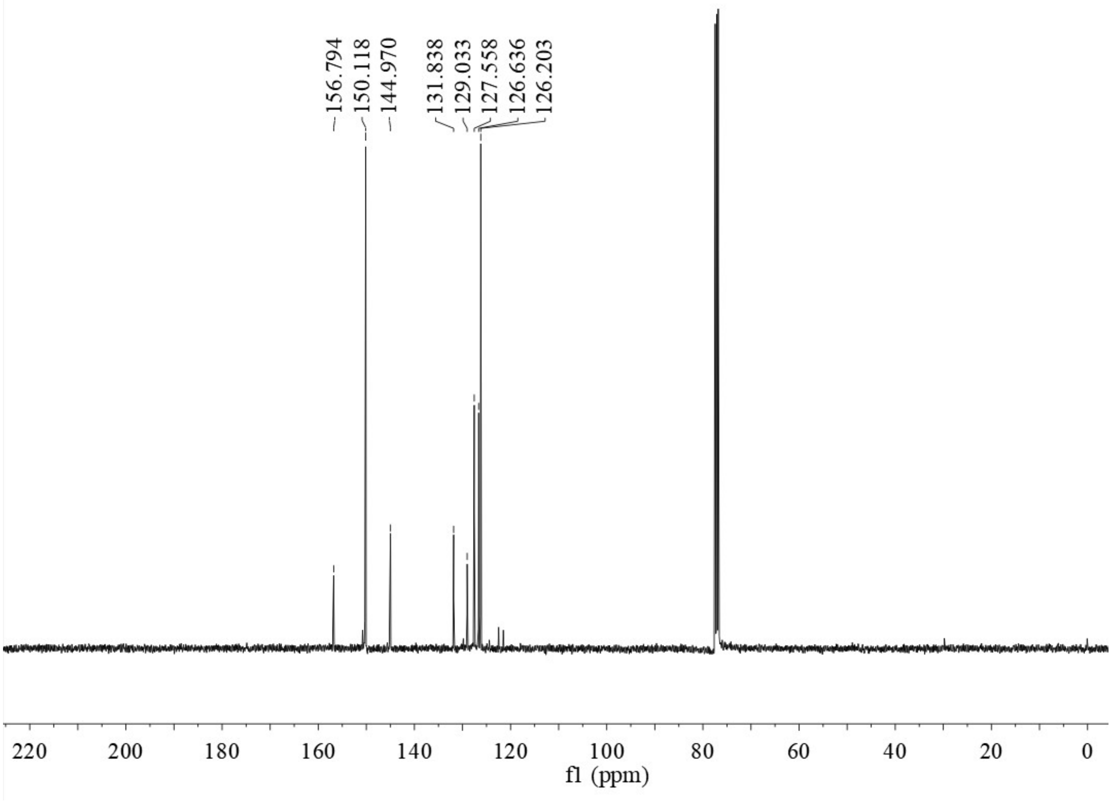

Figure S30. [1]13 NMR spectrum of PNS in $CDCl_3$。

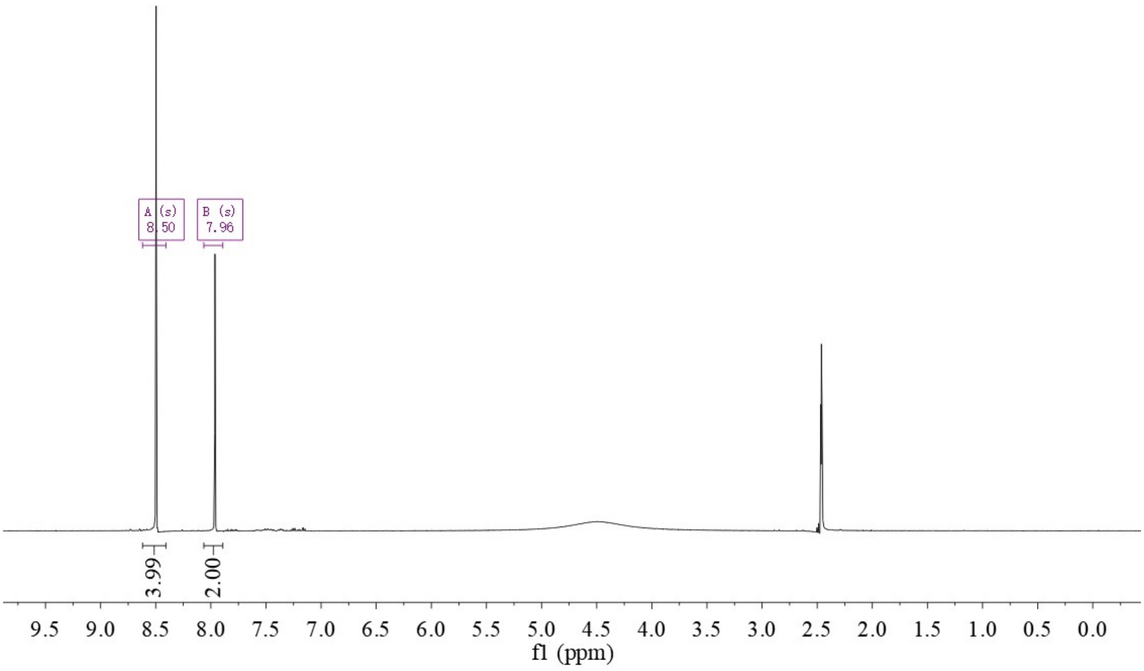

Figure S31. ^{13}H NMR spectrum of DPBT in DMSO-d_6.

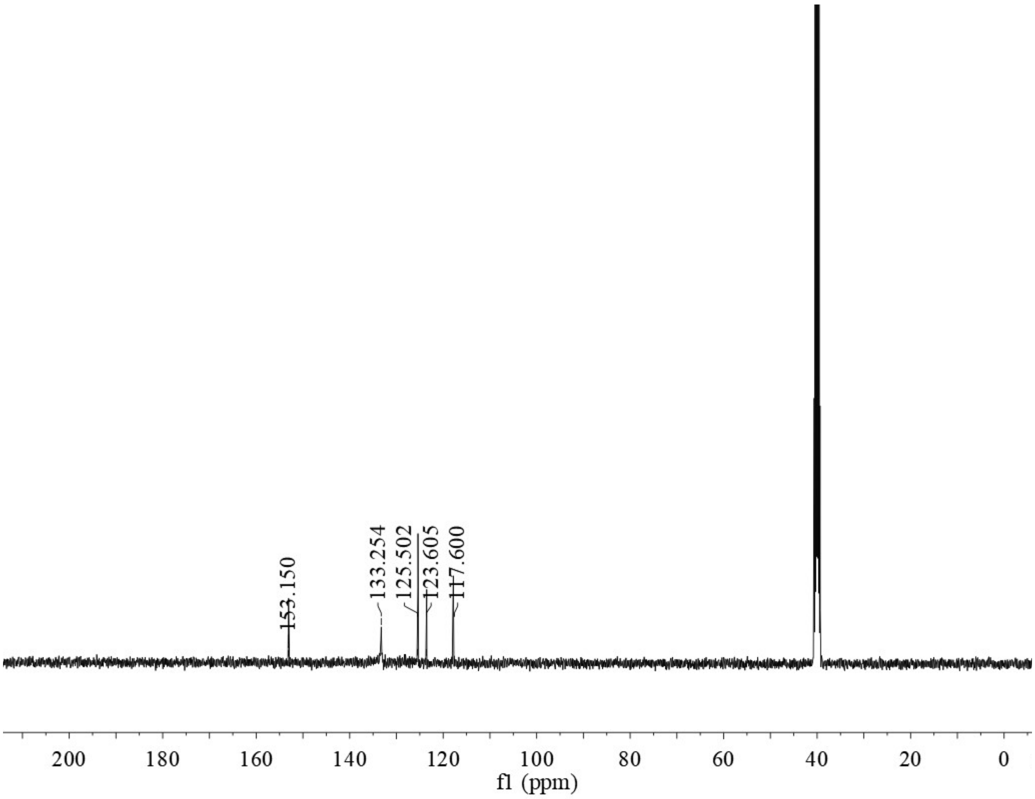

Figure S32. ^{13}C NMR spectrum of DPBT in DMSO-d_6.

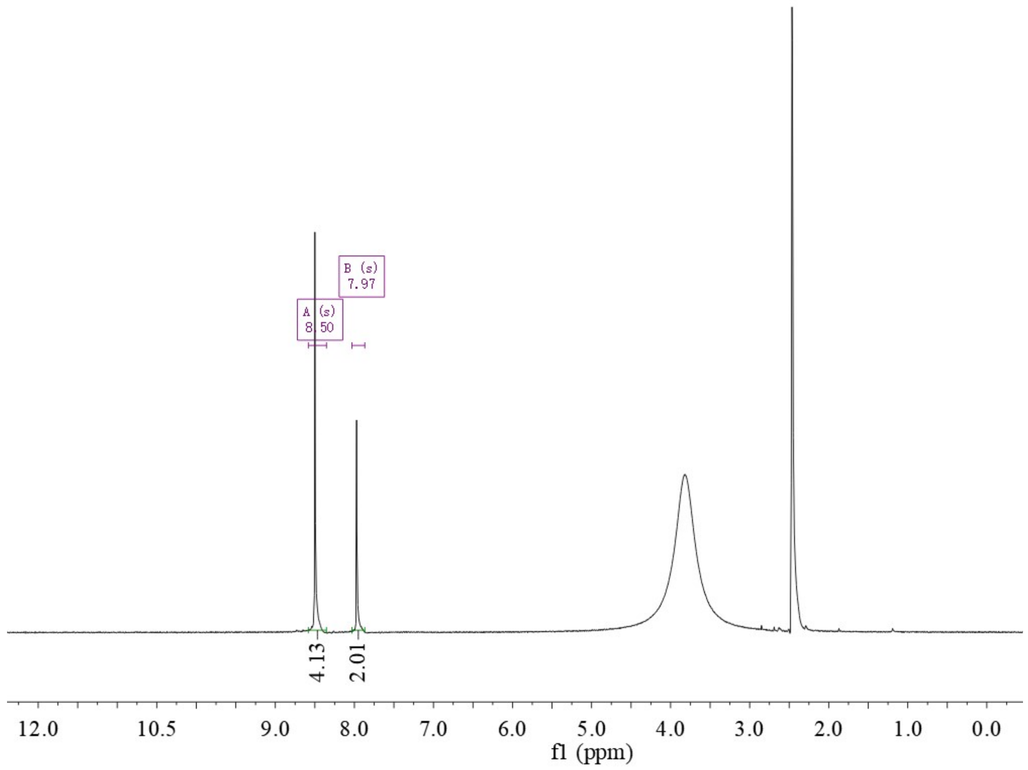

Figure S33. ^{1}H NMR spectrum of DPBS in DMSO-d_6.

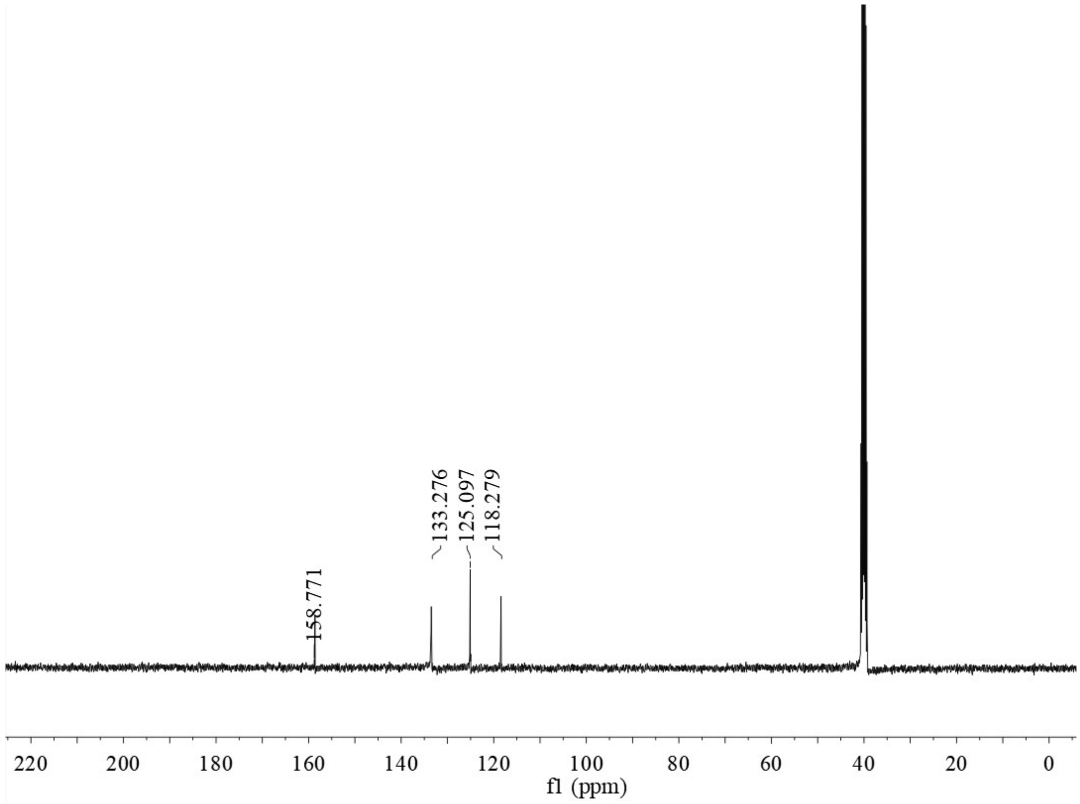

Figure S34. ^{13}C NMR spectrum of DPBS in DMSO-d_6.

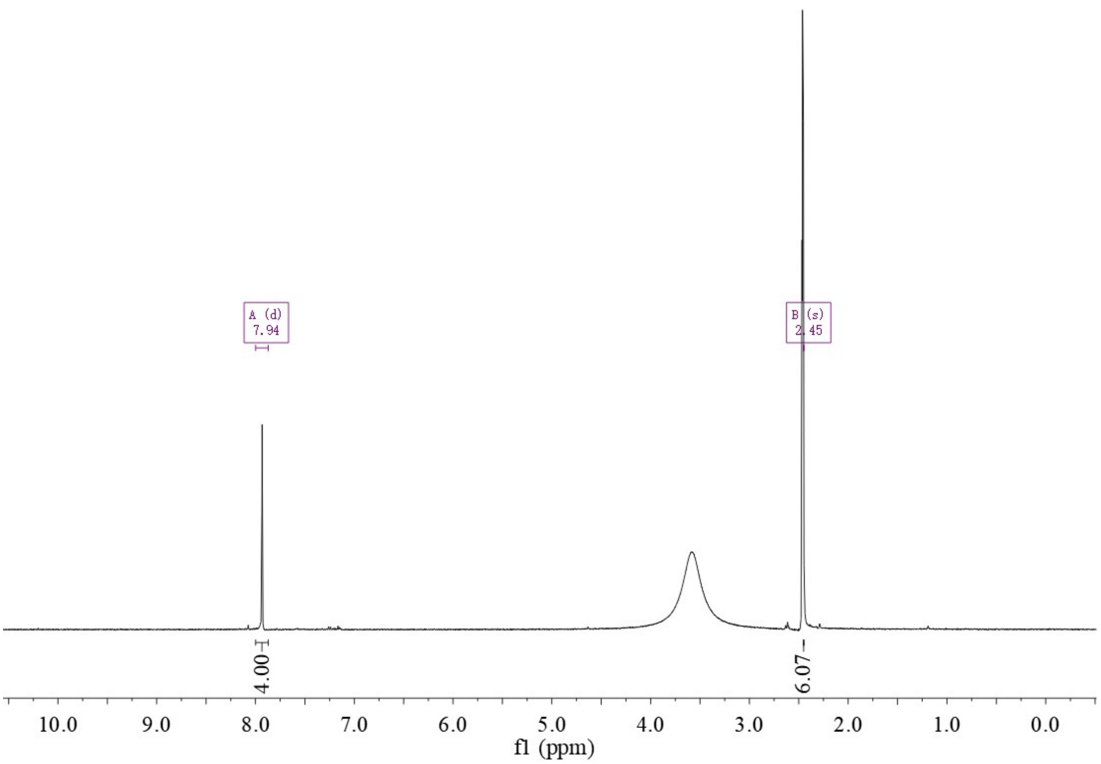

Figure S35. ^{1}H NMR spectrum of DDPBT in DMSO-d_6.

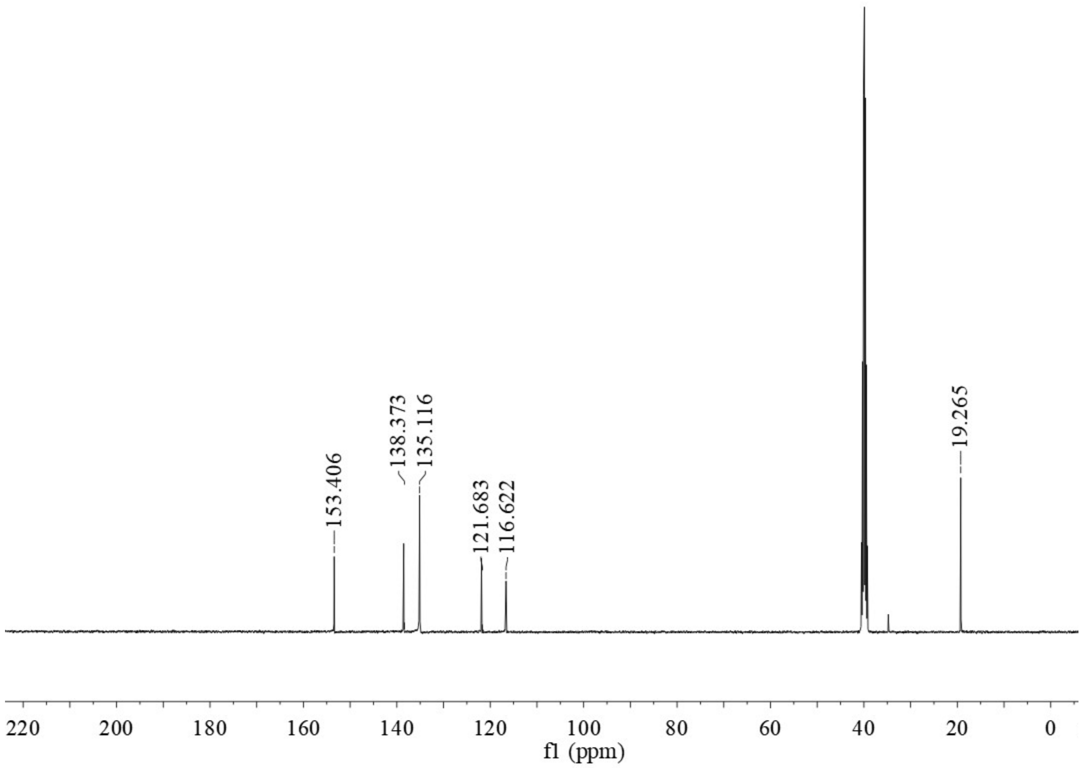

Figure S36. ^{13}C NMR spectrum of DDPBT in DMSO-d_6.

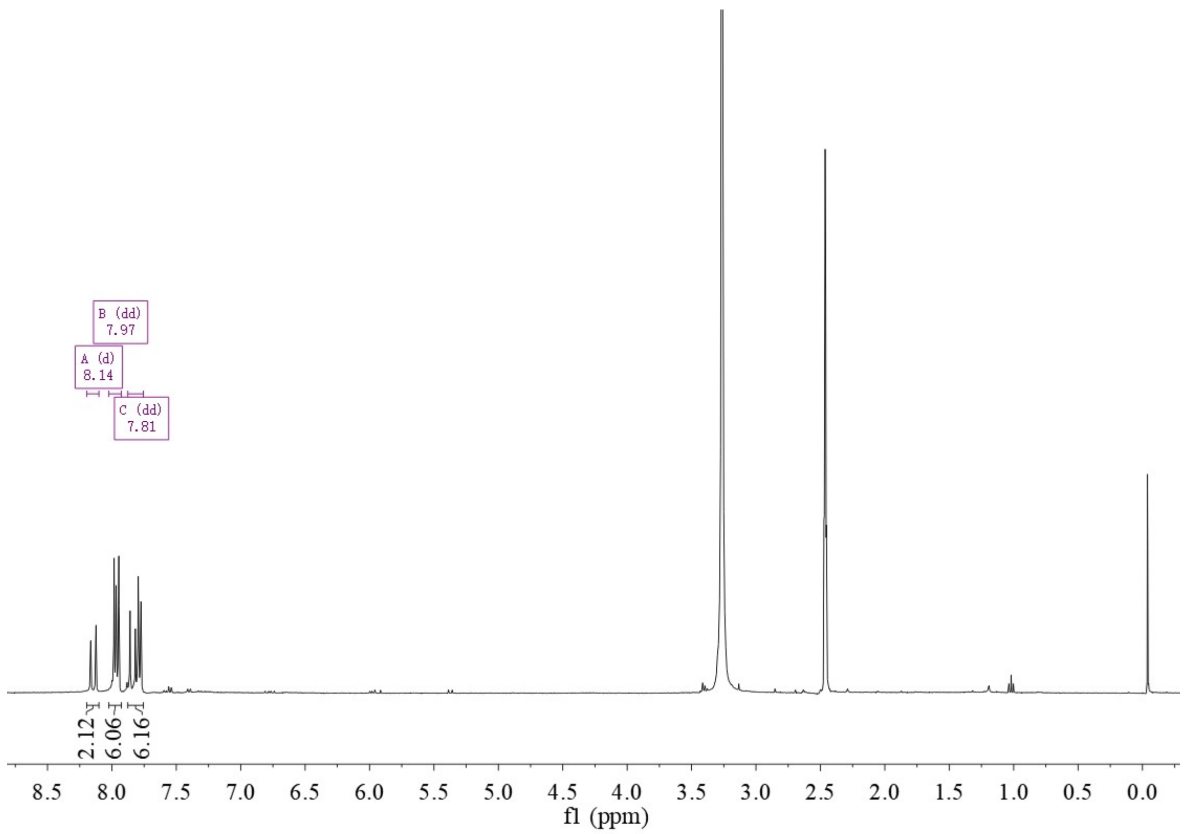

Figure S37. ^{1}H NMR spectrum of BTEBA in DMSO-d_6.

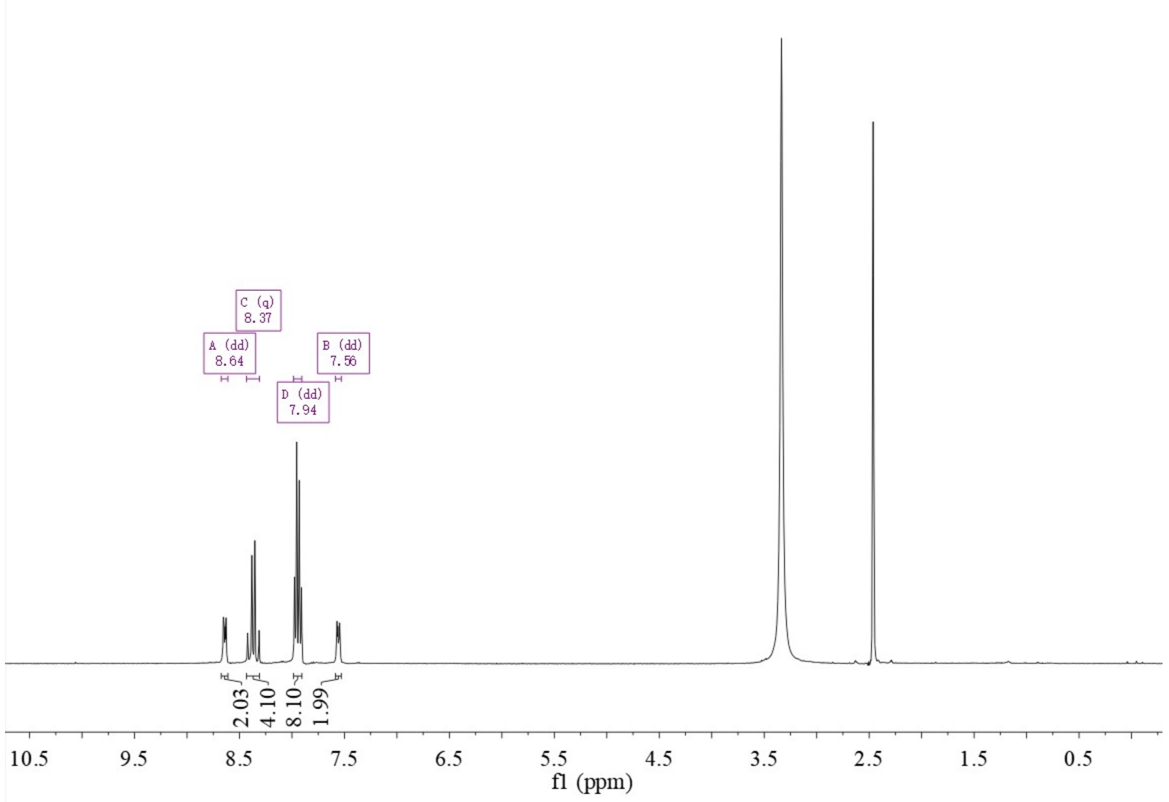

Figure S38. ^{1}H NMR spectrum of NTEBA in DMSO-d_6.

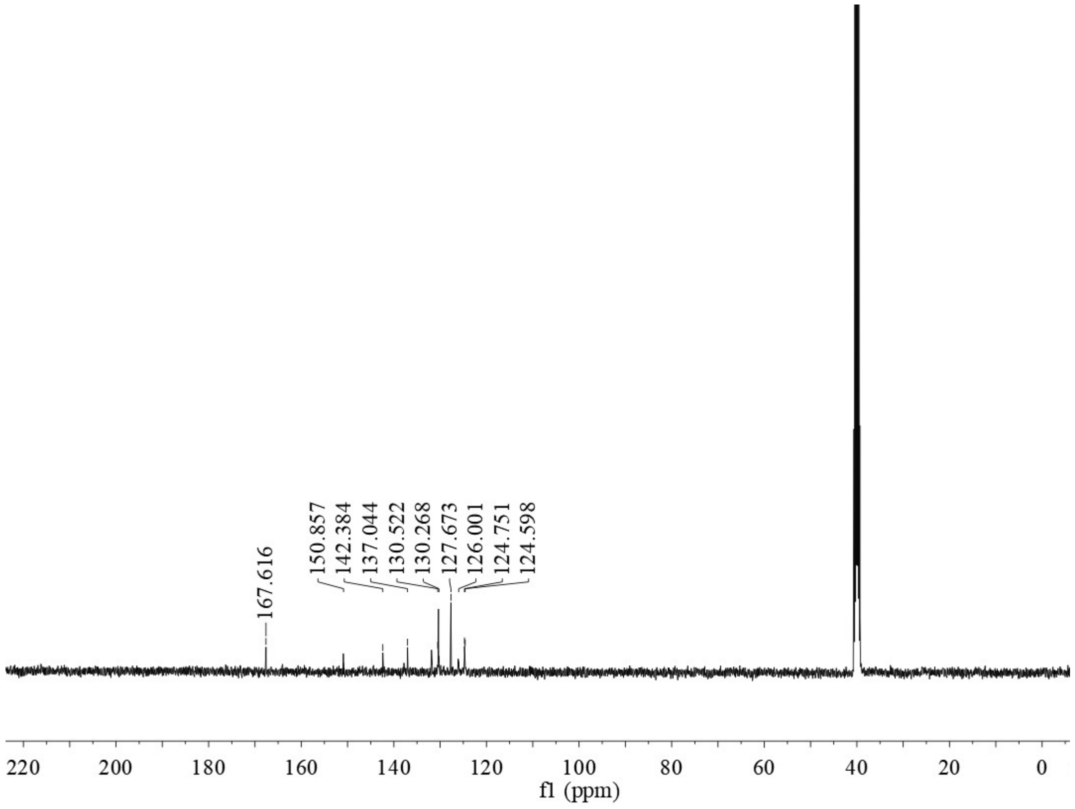

Figure S39. ^{13}C NMR spectrum of NTEBA in DMSO-d_6.

References

1. Ams, M. R.; Trapp, N.; Schwab, A.; Milić, J. V.; Diederich, F., Chalcogen Bonding "2S–2N Squares" versus Competing Interactions: Exploring the Recognition Properties of Sulfur. *Chem. Eur. J.* **2019,** *25* (1), 323.

2. Idris, I.; Tannoux, T.; Derridj, F.; Dorcet, V.; Boixel, J.; Guerchais, V.; Soulé, J.-F.; Doucet, H., Effective modulation of the photoluminescence properties of 2,1,3-benzothiadiazoles and 2,1,3-benzoselenadiazoles by Pd-catalyzed C–H bond arylations. *J. Mater. Chem. C* **2018,** *6* (7), 1731.

3. Wu, S.; Ren, D.; Zhou, K.; Xia, H. L.; Liu, X. Y.; Wang, X.; Li, J., Linker Engineering toward Full-Color Emission of UiO-68 Type Metal-Organic Frameworks. *J. Am. Chem. Soc.* **2021**, 143 (28), 10547-10552.

4. (a) Lee, C. T.; Yang, W. T.; Parr, R. G. Development of the Colle-Salvetti Correlation-Energy Formula into a Functional of the Electron Density. *Phys. Rev. B* **1988**, *37*, 785. (b) Becke, A. D. A new mixing of Hartree-Fock and local density-functional theories. *J. Chem. Phys.* **1993**, *98*, 1372. (c) Becke, A. D. Density-Functional Thermochemistry. III. The Role of Exact Exchange. *J. Chem. Phys.* **1993**, *98*, 5648. (d) Stephens, P. J.; Devlin, F. J.; Chabalowski, C. F.; Frisch, M. J. Ab Initio Calculation of Vibrational Absorption and Circular Dichroism Spectra Using Density Functional Force Fields. *J. Phys. Chem.* **1994**, *98*, 11623.

5. Frisch, M. J. et al. *Gaussian 09*, rev. D.01; *Gaussian Inc.*: Wallingford, CT, **2013**.

6. Dolomanov, O.V., Bourhis, L.J., Gildea, R.J, Howard, J.A.K. & Puschmann, H. OLEX2： A Complete Structure Solution, Refinement and Analysis Program. *J. Appl. Cryst.* **2009**, *42*, 339.

7. Sheldrick, G.M. SHELXT-Integrated Space-Group and Crystal-Structure Determination. *Acta Cryst*. **2015**, A71, 3.

8. Sheldrick, G.M. Crystal Structure Refinement with SHELXL. *Acta Cryst*. **2015**, C71, 3-8.

9. Spek, A. L., Single-crystal structure validation with the program PLATON. J. Appl. Cryst. **2003**, 36, 7-13.
